# Martini on the Rocks: Can a Coarse-Grained Force Field Model Crystals?

**DOI:** 10.1021/acs.jpclett.4c00012

**Published:** 2024-01-23

**Authors:** A. Najla Hosseini, David van der Spoel

**Affiliations:** Department of Cell and Molecular Biology, Uppsala University, Box 596, SE-75124 Uppsala, Sweden

## Abstract

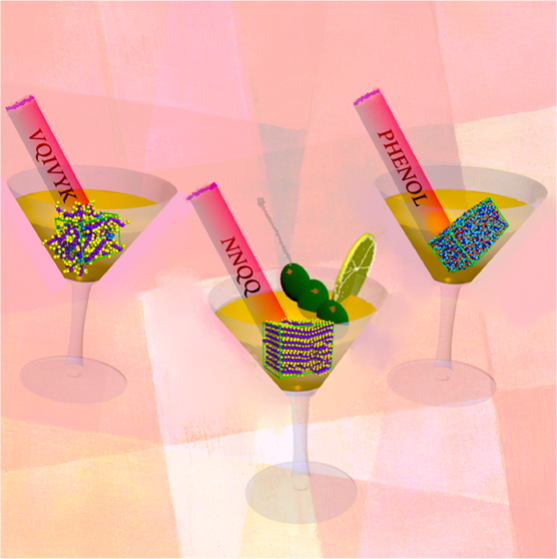

Computational chemistry is an important tool in numerous scientific disciplines, including drug discovery and structural biology. Coarse-grained models offer simple representations of molecular systems that enable simulations of large-scale systems. Because there has been an increase in the adoption of such models for simulations of biomolecular systems, critical evaluation is warranted. Here, the stability of the amyloid peptide and organic crystals is evaluated using the Martini 3 coarse-grained force field. The crystals change shape drastically during the simulations. Radial distribution functions show that the distance between backbone beads in β-sheets increases by ∼1 Å, breaking the crystals. The melting points of organic compounds are much too low in the Martini force field. This suggests that Martini 3 lacks the specific interactions needed to accurately simulate peptides or organic crystals without imposing artificial restraints. The problems may be exacerbated by the use of the 12-6 potential, suggesting that a softer potential could improve this model for crystal simulations.

The structure of a protein is defined, in principle, by inter-residue hydrogen bonds providing specificity in combination with well-packed side chains of aliphatic or aromatic character, providing thermodynamic stability through the hydrophobic effect.^1^ Indeed, it has been suggested that the structure of a protein is to a large extent governed by its interaction with the solvent, water.^2^ Amyloid peptide fibrils are an intriguing example of these structural features, containing β-sheets in one plane and tightly packed side chains perpendicular to it.^3−5^ Amyloid peptide fibrils such as yeast prion protein Sup35, insulin, Alzheimer’s amyloid-β, τ, and amylin have pairs of tightly bound β-sheets known as “steric zipper” structures.^3,4,6,7^ These structures run parallel to the fibril axis and play a crucial role in amyloid aggregations.^8^ The stability of these peptide structures is influenced by factors such as hydrogen bonds along the fibril axis, van der Waals interactions, electrostatic interactions, the hydrophobic effect, and π–π stacking between side chains.^9−11^

The elucidation of the molecular structure of amyloid fibrils has been a challenge due to the inherent difficulty in generating well-diffracting crystals.^12^ Early structural investigations of amyloid fibrils hence focused on short polypeptides, such as GNNQQNY and KLVFFAE, as they can be assembled *in vitro*, resulting in well-ordered fibers that are suitable for analysis using techniques such as X-ray diffraction, electron microscopy, and solid-state nuclear magnetic resonance (NMR).^13^ Over the years, fibril structures of amyloid proteins have been proposed on the basis of solid-state NMR^14^ and cryo-electron microscopy experiments,^15^ with X-ray diffraction analysis used for shorter amyloid peptides.^3^ Michaels and colleagues conducted a comprehensive investigation of the dynamics of oligomeric species during the aggregation of the amyloid-β42 peptide, using an approach combining theory, experiment, and simulation. Their findings demonstrate that, although mature amyloid fibrils stem from oligomers, a majority of amyloid-β42 oligomers dissociate into their monomeric forms rather than undergoing fibril formation and only a small subset of the oligomers undergo a transition to form fibrillar structures.^16^ To complement experimental investigations, computer simulations can offer valuable insights by providing molecular models of the biological process of amyloid aggregation. For instance, Ganguly et al. investigated the aggregation of τ fragments containing the VQIVYK and VQIINK segments and their mixture, using experimental techniques and replica exchange molecular dynamics simulations. Their findings indicate that the VQIVYKPVDLSK fragment has a higher propensity for aggregation than GKVQIINKKLDL, and they suggest that heterodimer interactions may be involved in initiating Tau aggregation.^17^ In another study combining experiments with simulations, Chen et al. determined that the aggregation-prone VQIVYK peptide, with its upstream sequence, forms metastable compact structures that influence its propensity for aggregation.^18^ Nguyen and co-workers reviewed additional amyloid simulation studies.^19^

The past two decades have seen the introduction and widespread adoption of coarse-grained (CG) force fields for molecular simulations.^20−23^ A recent review by Noid explains challenges and promising directions in the CG field.^24^ By grouping atoms together, we can reduce the total number of particles, and the computational cost of simulations is reduced drastically. If, for instance, four atoms are modeled as one particle, the cost is reduced by a factor of ∼4^2^. This promised the possibility of studying larger systems and/or using longer simulation times. Grouping of atoms can be done selectively, for instance, just for the solvent immersing a molecule of interest,^25^ or throughout the entire system. In this manner, degrees of freedom can be averaged out consecutively until a desired “resolution” is obtained.^26^ Although the word “resolution” is used ubiquitously in the CG modeling field,^27^ it is written in quotation marks here, to avoid confusion with the resolution obtained in experimental structural biology, which is a property of the data, rather than a model parameter.

In virtually all CG models, the energy function is replaced by a free energy function, which means force field parameters become dependent on temperature (although this is not a large problem in all cases^23,28^) and time becomes ill-defined because the “forces” derived from such a potential function include the derivative of entropy with respect to the particle positions.^29,30^ These models have been optimized to reproduce free energies starting from atomistic models; however, the reduced atomic detail leads to specific interactions being approximated. This strongly suggests that energy barriers will be decreased and kinetics overestimated. Some recent reviews describe the state of the art in CG simulations.^20,23,31^ Indeed, seeing that the potential energy surface actually describes free energies, it is unclear what ensemble is produced by CG “simulations”, but it is likely different from the ensemble of the atomistic simulations on which the coarse grained models are based.^32^

Seeing that projects are ongoing targeting modeling of entire cells or large virus particles, such as SARS-CoV-2, using CG force fields,^33−35^ we believe it is worthwhile to consider what predictive power such models could contribute. Attempts to model dense protein solutions or even the *Escherichia coli* cytoplasm with all-atom (AA) force fields were sobering in that they demonstrated the complexity of such undertakings and shortcomings on the part of the physical models.^36−38^ It is therefore questionable whether studies of large complex biological systems would fare better using less-detailed models. Indeed, a recent study of mechanoporation of biological membranes shows that the Martini 3 force field yields a reasonable structural model but that under pressure pores are formed faster than with a corresponding AA force field.^39^ Moreover, Martini 3 has also been found to have other issues, such as underestimation of the radius of gyration of intrinsically disordered proteins by ≈30% and the overestimation of protein–protein interactions when compared to small-angle X-ray scattering (SAXS) data,^40^ incorrect prediction of coiled-coil dimer structures, short transmembrane peptides that were not stable inside a membrane,^41^ and a failure to insert transmembrane helix dimer proteins into dodecylphosphocholine micelles.^42^ A recent study by Sasselli and Coluzza to model short peptide self-assembly highlights challenges arising from overestimated hydrophilicity due to charged termini and disruptions in π stacking interactions when using Martini 3.^43^ It has also been shown that Martini fails to accurately capture the enthalpy–entropy decomposition for pair correlations in bilayers and that it leads to unusual helix–helix attractions in bilayers and exhibits unphysical fluctuations at intermediate length scales for lipid bilayers.^44^ We do note that simulations using the UNRES coarse-grained model have provided insight into the conformational changes leading to amyloid-β fibril formation,^45,46^ although the time scales covered remain short.

Clearly, the development of force fields necessitates an independent assessment of such models. The study of crystal lattices has been a key component in the history of molecular dynamics, and such studies have proven to be useful in investigating the quality of underlying force fields, establishing correlations between simulated ensembles and experimental structure factors.^47,48^ Hence, in this work, we investigate whether the Martini 3 model can reproduce properties of crystals consisting of amyloid peptides and organic compounds that we have studied previously using AA models.^49,50^ We present CG simulations of 12 amyloid peptide crystals at cryo temperature and the temperature used for growing the crystals and evaluate the stability of the crystal. To do so, we have performed crystal peptide simulations considering the “bare” Martini 3 (M3) force field, a variant including side chain restraints (M3′)^51^ and a variant including both side chain restraints and restraints on the intramolecular secondary structure (M3″).^52^ Two different water models were considered: regular W for both M3 and M3′ and tiny water (TW) for the M3″ force field model. In addition, we determined the melting points of organic crystals, providing a quantitative measure of the accuracy of the Martini 3 force field in comparison to the experimental data and AA models.

The kinetic stability of crystals of 12 peptides in CG simulations using three Martini 3 variants (M3, M3′, and M3″) was evaluated visually (Figure 1) and by evaluating lattice parameters (Figures S1–S12). Table 1 shows the average deviation from the experimental crystals of the box edge, angle, and volume for the peptides at temperatures corresponding to crystal growth and data collection (cryo temperature). In the case of the M3 model (Figures S1–S4), a comparison of the lattice size of peptides during the simulations shows better stability of GNNQQNY and NNQQNY using the Berendsen barostat than the Parrinello–Rahman barostat. Some peptides, such as GNNQQNY and NNQQNY, keep the cell edges stable over the simulation time after an initial change using the Berendsen barostat. The β and γ unit cell angles for LYQLEN and VQIVYK are both unstable at 310 and 291 K, respectively, using the Parrinello–Rahman barostat. These peptides display rapid and large changes for all cell edges. However, using the Brendsen barostat, they show better stability using this model. The cell shapes for all peptides using the Berendsen barostat, except GNNQQNY and NNQQNY, change rapidly at room temperature as evidenced from both the length of the supercell edges and the lattice angles. Indeed, MVGGVV (1) shows unstable α, β, and γ angles at 298 K.

**Figure 1:**
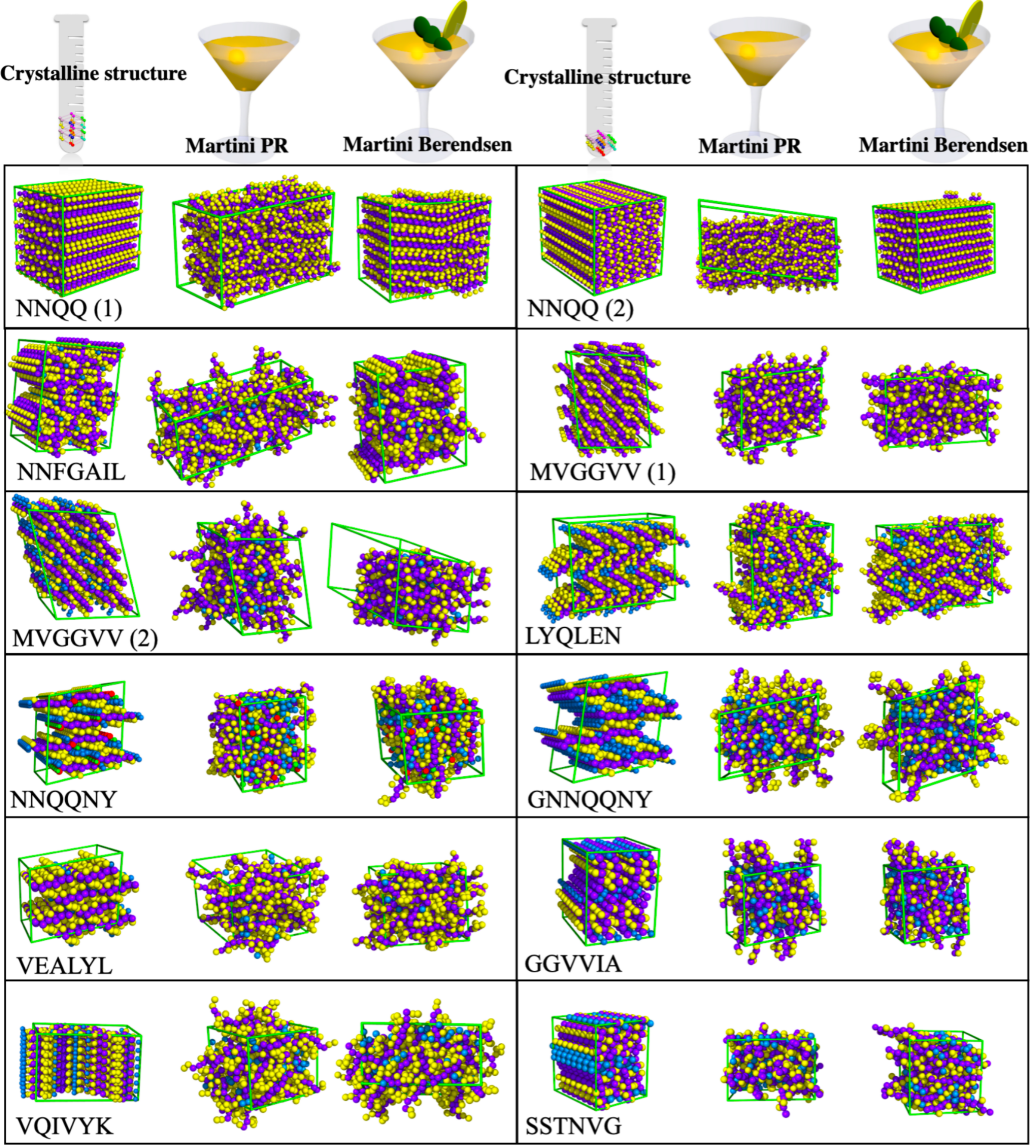
Crystals of 12 amyloid peptides before and after a 200 ns simulation at room temperature with a modified Martini 3 (M3″) force field using tiny water beads using the Parrinello–Rahman (PR) or Berendsen (B) barostat.^53^ Purple for the backbone, yellow for side chain beads, and cyan for crystal water molecules. Zinc and acetate beads associated with the NNQQNY peptide are colored red and green, respectively. For NNQQ as well as MVGGCC, two different crystal structures were used as a starting point for the simulations. See ref (49) for details.

**Table 1:** Mean Absolute Deviations from Lattice Parameters for the AA Force Fields, AMBER19SB, CHARMM36m, and OPLS-AA/M (simulations from ref (49)) and Martini 3 Force Fields (this work) as a Function of Temperature [CT, cryo temperature; RT, room temperature (see Methods)]^a^

		AMBER	CHARMM	OPLS	M3	M3	M3′	M3′	M3″	M3″
property	*T* (K)	B	B	B	PR	B	PR	B	PR	B
box edge (%)	CT	0.2	0.3	0.1	15.5	3.7	12.3	3.7	8.5	3.3
	RT	0.4	0.1	0.4	22.4	2.4	20.6	3.2	16.4	2.8
angle (deg)	CT	0.2	0.2	0.1	2.9	0.2	2.1	0.9	3.2	1.0
	RT	0.3	0.2	0.3	8.6	0.8	5.0	0.7	3.8	0.8
volume (%)	CT	0.3	1.0	0.3	17.8	17.0	12.8	8.9	9.7	7.9
	RT	0.9	0.1	0.5	13.1	8.4	12.9	6.8	7.3	6.1

a The barostat used is indicated as PR (Parrinello–Rahman) or B (Berendsen). M3, M3′, and M3″ indicate original Martini 3, Martini 3 with side chain corrections, and Martini 3 with side chain and secondary structure corrections, respectively.

M3′ (Figures S5–S8) and M3″ (Figures S9–S12) demonstrate a more stable trend, even in the presence of a significant deviation from the experimental structure, when compared to the M3 model.

Our simulations indicate that M3″ using the Berendsen thermostat yields the most stable simulations; however, a deviation of 6–8% in the volume of the crystal with respect to the native structure is observed (Table 1). Individual box edges may change by ≤100% in the simulation. An example of such an issue is NNFGAIL at 293 K, demonstrating very rapid change using both Parrinello–Rahman (Figure S9) and Berendsen (Figure S11) barostats. In the Martini 3 paper,^27^ it is suggested that the particle mesh Ewald (PME^55^) method may be needed to handle long-range electrostatic interactions for some systems. However, when comparing lattice sizes and angles at both 293 and 100 K, we find that this algorithm does not lead to more stable results either (Figures S13–S16).

To obtain a more quantitative description of the transformation of peptides from a crystalline to a liquid state in simulations using Martini 3, we calculated the radial distribution function (RDF) for the NNQQ1 peptide, which does not contain any water molecules in its crystal. The RDFs (Figures S19–S22) were computed on the basis of the backbone beads of the peptides, loosely corresponding to the hydrogen bond distance. In the case of the M3 model, there is no difference between Berendsen and Parrinello–Rahman barostats and the crystal undergoes a transition to a liquid state, eventually melting. A comparison between the RDF plots of Berendsen and Parrinello–Rahman barostats shows better stability with Berendsen, considering the M3′ and M3″ force fields at cryo temperature. However, the shift in the calculated RDF and the decrease in RDF height in the simulations, compared to the initial RDF at both cryo and room temperatures, confirm the melting of the peptide and transformation to a liquid state using both Parrinello–Rahman and Berendsen barostats for all three Martini 3 variants (Figures S19–S22). In contrast, an example RDF from our previous AA study using CHARMM36m^49^ shows that the hydrogen bond structure is maintained in AA simulations (Figure S23).

Compared to our previous work using three atomistic force fields, AMBER19SB, CHARMM36m, and OPLS-AA/M,^49^ a much larger deviation of lattice parameters is observed for virtually all peptides using both the Parrinello–Rahman (which is the recommended barostat in Martini 3) and the Berendsen barostat (which is more resistant to fluctuations). The MVGGVV (1) crystal is destabilized and deformed in all M3, M3′, and M3″ models in a manner similar to that of AMBER19SB, CHARMM36m, and OPLS-AA/M force fields at room temperature, but to a larger degree. Even at cryo temperature, very rapid changes in angles and edges occur for this peptide. In all cases, there is a large difference between the results from CG and AA simulations. Most of the peptide crystal supercells in the Martini simulations are unstable, in contrast to the AA. Table 1 shows that the deviation from the crystal lattice parameters is more than an order of magnitude larger using the Martini 3 force field than in the AA force fields. The comparisons between Parrinello–Rahman and Berendsen barostats suggest that better stability is obtained with the use of the Berendsen barostat in Martini simulations. However, it should be noted that even with this choice and addition of intramolecular restraints (M′ and M″), stability issues persist (Figures S3–S12).

We now turn our attention to molecular crystals. Previous simulation work using an all-atom force field has shown that it is difficult to model crystals of organic molecules (Figure 2), because these often have only weak interactions.^50^ It is well established that detailed force field models are needed to estimate melting points,^56^ and for organic compounds, a root-mean-square deviation of ∼40 K was found comparing AA simulations to experimental numbers.^50^ Melting points were computed using the solid–liquid coexistence method^57^ and determined from the diffusion constant as a function of temperature (Table 2 and Figures S24–S47). There is no or very poor correlation between experimental melting points and those obtained from Martini 3 simulations (Figure S50). Upon analysis of the temperature at which the diffusion constant is zero, even glassy states may be counted as solid. To investigate this more in detail, we calculated the radial distribution function for pyridine and phenol at 5 K (Figures S48 and S49). Even at this low temperature, one can see that the crystals turn into a glassy state using Martini. Table 2 shows that the Martini 3 model even with this generous definition of the solid state yields melting points that are considerably lower than the experimental data or those from AA simulations. The root-mean-square deviation from experiment is almost 5 times higher for the CG than the AA model. Upon more detailed analysis, it becomes evident that some of the one-bead compounds persist in the crystalline form, although not necessarily matching the atomistic crystal structures. Meanwhile, most of the larger compounds undergo melting (Figure 2 and Table 2).

**Figure 2:**
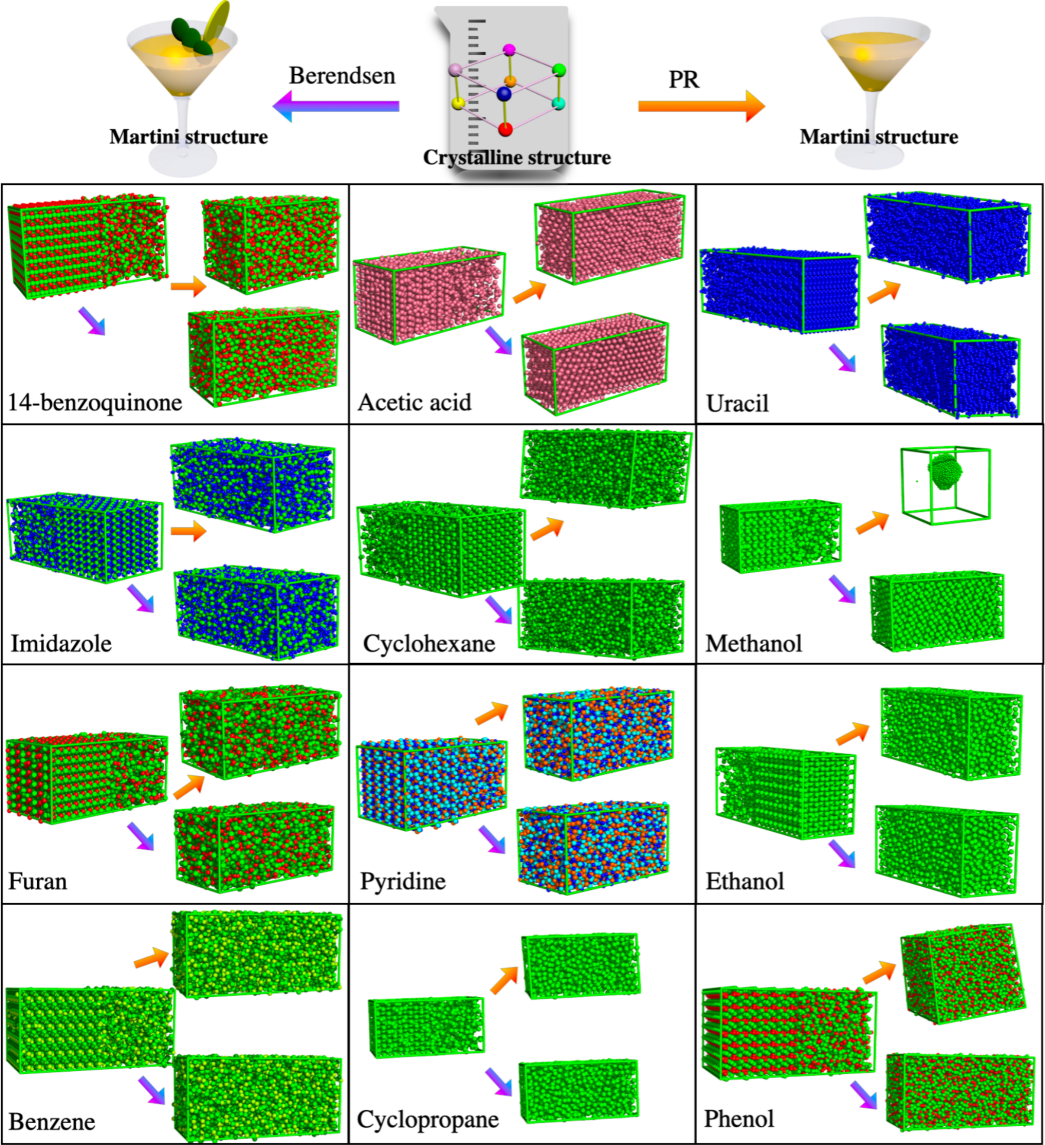
Solid–liquid coexistence simulation systems of 12 organic crystals using the Martini 3 force field. Structures before and after simulation at 180 K, using the Parrinello–Rahman^54^ (PR, orange arrow) or Berendsen^53^ (purple arrow) barostat.

**Table 2:** Small Molecules Are Simulated in the Crystalline State^a^

			*T*_melt_ (K)			
				GAFF	Martini	
compound	#P	#M	exptl	B	PR	B
1,4-benzoquinone	4	1152	403	388	140	140
acetic acid	1	1536	290	294	181	181
benzene	3	1536	279	250	5	80
cyclohexane	2	1260	280	302	100	120
cyclopropane	1	840	175	118	120	130
ethanol	1	1600	159	189	150	170
furan	3	1024	188	130	50	80
imidazole	3	1680	364	278	60	100
methanol	1	1024	176	186	0	240
phenol	3	1680	314	279	20	100
pyridine	3	1440	232	246	5	80
uracil	5	1400	611	748	100	200
RMSD				55	247	198

a The number of Martini particles (#P) per molecule and the number of molecules (#M) in the crystals.^58^ Experimental^59^ and simulated melting points. Melting temperatures (*T*_melt_) for the generalized Amber FF (GAFF^60^) from ref (50). Martini from this work. The *T*_melt_ is defined here as the lowest temperature for which the diffusion constant deviates from zero (Figures S24–S47). The pressure scaling algorithm used is indicated for the results from simulations. The last row gives the root-mean-square deviation (RMSD) in kelvin from the experiment for both models. The PR (Parrinello–Rahman) or B (Berendsen) barostat was used.

Careful benchmarking of methods used in AA force field simulations has led to a reasonable understanding of the merits of the available models. The quantitative evaluation of accuracy is easiest for small (organic) compounds, for which standard methods for force field parametrization are readily available^60−64^ and for which a large body of data is available from experiments^65,66^ or from high-quality quantum chemistry.^67^ Examples of such benchmarks are available for the gas phase,^50,68,69^ the liquid phase,^70−74^ and the solid phase.^50,74^ These studies have established the root-mean-square errors for predictions from atomistic force fields to be ∼7 kJ/mol for enthalpies of vaporization,^70,71^ ∼3% for liquid densities,^70,71^ 8 kJ/mol for enthalpies of sublimation, 5% for solid densities, and 40 K for the melting point.^50^ We recently extended this benchmarking work to a study of peptide crystals.^49^ In that study, we performed simulations of 12 peptide crystals using three modern atomistic force fields and found that some of the crystals deformed during long simulations (Table 1). From this, we concluded that simulations of organic crystals and peptides are challenging for atomistic force fields. Nevertheless, it may be possible to study amyloid peptide crystals and fibrils with suitable adaptations and refinement of atomistic force fields.^75^ Time scales for fibril formation present another hurdle on the road. Sarthak and colleagues attempted to explore the effects of point mutations on protein condensates, which have implications in neurodegenerative disorders, but even using the most powerful Anton-2 computer,^76^ their simulations were unable to reach the time scales required for fibril formation *in vitro*.^77^

Buell emphasizes that the thermodynamic aspects of fibril formation are an important topic for future amyloid research.^7^ Because aggregation of proteins and large peptides is slow, shorter peptides can still be useful for gaining a deeper understanding of the aggregation process. For instance, the potential of mean force calculations of crystal formation could help to shed light on amyloid thermodynamics,^75^ and such calculations should be tractable for all-atom models. Teijlingen et al. have investigated the self-assembly of short peptides using different Martini force fields (3/2.1/2.1P/2.2/2.2P).^78^ They highlighted the challenge in comparing results between Martini 2.1 and Martini 3 due to differences in bead types (side chain and backbone) and different Lennard-Jones terms for the same beads. One issue they reported is the overstabilization of the “π-stacking” effect in Martini 2.1, yielding an energy minimum of −21.0 kJ/mol, compared to high-level quantum chemistry calculations in the gas phase that give values of −7.5 to −11.7 kJ/mol. On the contrary, Martini 3 produces a more accurate “π-stacking” energy of −12.7 kJ/mol. However, they obtained a stack of nanodiscs instead of the expected tubular structure, highlighting issues with the correct packing of compounds.^78^

One of the key motivations for simulating crystal structures is to evaluate and test force field parameters. Indeed, crystals have long served as a crucial testing ground for the development of simulation models.^47^ The main point of this work is to evaluate the packing properties in a crystalline system. To do so, simulations of peptides were performed using three Martini 3 variants (see Methods for details). A comparison between the results obtained with the two barostats indicates that the kinetic stability is higher when using the Berendsen barostat for both peptides and organic crystals. Despite this choice and the added intramolecular restraints in M′ and M″, stability issues persist. Because peptides are short, the additional stabilization due to the elastic bond corrections is more limited than, for example, for a protein. The corrections used span the whole length of the peptide, which means we enforce the β-strand to remain in the same conformation. In combination with the side chain restraints, the peptides become almost entirely rigid. Nevertheless, the peptide crystals deform significantly (Figure 1, Figures S5–S8, and Table 1), and molecular crystals are unstable, as well (Figure 2 and Table 2). One can see from radial distribution functions that the packing interactions are not sufficiently strong for peptide backbones (Figures S19–S22) or organic compounds (Figures S48 and S49) to maintain stable crystals in CG simulations using Martini 3. Our findings, derived from an exploration of three force field variants, coupled with the implementation of different barostats and treatment of long-range electrostatics, underscore the limitations of the force field in maintaining accurate packing.

It is self-evident that large and complex simulation systems need a long time to equilibrate. For instance, for the small satellite tobacco necrosis virus it was found that atomistic simulation for 1 μs was not enough for the virus capsid to relax.^79^ It therefore seems unfounded to draw conclusions about the “fast dynamics” of components of the much larger SARS-CoV-2 virus system on the basis of a short 500 ns Martini 3 simulation,^35^ not in the least because increased kinetic rates are a “feature” of CG models in general.^32^ Both the mentioned dynamics of membrane-embedded proteins and the diverse binding preferences of certain lipid types for specific sites of the membrane-embedded proteins under these conditions could potentially be attributed to model artifacts.

It is well-established that the Lennard-Jones 12-6 potential^80^ has a repulsion that is too steep for atomistic simulations.^81−89^ When data are averaged over atomic interactions, CG beads necessarily have to become larger than atoms to maintain the correct densities. It would seem logical to make the beads softer to allow for more flexible interactions, and it is curious that the Martini developers continued using the Lennard-Jones 12-6 potential for their models. This potential is the reason that most crystals crack at once in Martini simulations (Figures 1 and 2). Moreover, through the elimination of detailed atomistic interactions that give rise to friction to motion, CG models become less viscous, making the interpretation of CG dynamics challenging.^23,29,90^ To improve Martini to be able to model crystals, the explicit introduction of electrostatic interactions could be considered.^91^ Including electrostatic interactions allows for additional control over the properties of molecular systems. For instance, by artificially scaling the charges of the components in a CG model of an ionic liquid (IL), Saeielli and Wang were able to completely change the phase behavior of the IL.^92^ Alternatively, directional potentials could help to stabilize hydrogen-bonded structures like amyloid peptides.^93,94^ Within the crystal structure prediction community, work is ongoing to include anisotropic atoms to predict the relative energies of crystal polymorphs,^95^ strongly suggesting that models with simplified descriptions of the physics^48^ simply lack the detail needed to model peptide or organic crystals. Indeed, Strödel argues that more, not less, detail will be needed to address the biophysics of amyloid formation.^75^

## Methods

The initial structures of amyloid peptides in a CG representation for unit cells were generated using the Martinize2 Python script.^52^ Subsequently, we constructed supercells using the genconf tool in GROMACS, following a procedure similar to that described in our prior work. Files are available for inspection on our GitHub repository.^58^ Because the Martinize script does not generate water molecules, we used the existing atomistic supercell structures (oxygen atoms of water molecules) and incorporated them into the supercells. Then, we considered two available water models in the Martini 3 force field: regular (four water molecules modeled as a single Lennard-Jones site) and tiny beads (two water molecules as a single Lennard-Jones site) for mapping water molecules. After creating supercells, we removed three waters from every four closest waters (averaging the positions of identical neighbor water molecules interacting with the peptide) for the regular model and one water from every two closest waters for the tiny model. Topologies for peptides were generated by using the Martinize2 Python script for unit cells. We performed simulations with three different models: the original Martini 3 with regular water beads (M3), simulations with side chain fixation with regular water beads (M3′), and simulations with restrained side chains and simultaneous enforcement of the secondary structure for the peptides with tiny water beads (M3″).^27,52^

To conduct CG simulations using GROMACS,^96^ the systems were minimized with the steepest descent algorithm, followed by a constant-pressure equilibration (*NpT*) and a slow pressure coupling time of 1000 ps. Position restraints with a force constant of 1000 kJ mol^–1^ nm^–2^ were applied to the backbone of peptides during equilibration and were released for production. The temperatures were coupled to a V-rescale thermostat,^97^ which was set at crystal growing temperature (see ref (49) for details) and cryo temperature (100 K), with a coupling constant of 0.1 ps. The pressure was coupled to an anisotropic Parrinello–Rahman barostat^98^ with a coupling constant of 1000 ps, and the reference pressure set to 1 bar. We performed all simulations using the Berendsen barostat^53^ with a 1000 ps coupling constant, as well, as it dampens fluctuations by design.

Long-range electrostatic interactions were treated using a reaction field^99^ with a relative dielectric constant of 15^27^ or using PME.^55^ A neutral N-terminus was used for the positively charged peptide VQIVYK, using the Martinize2 script. A neutral C-terminus was used for peptides with negatively charged LYQLEN and VEALYL side chains. NNQQNY contains zinc and acetate; hence, we used a neutral N-terminus for this peptide. A zinc ion is not available in the Martini force field, so we used calcium beads but changed the mass to 65.38. To update the neighbor list, the Verlet neighbor search algorithm was used.^100^ Production trajectories were generated for 200 ns with cutoffs of 1.1 nm for the Lennard-Jones potential; no corrections were made for long-range van der Waals interactions.

A database of small molecule topology files for Martini 3 is provided by Alessandri et al.^101^ There are 12 shared compounds between the published Martini compounds and the data set published by Schmidt et al. (Table 2). Initial crystal structures were produced from the all-atom crystals that are available from GitHub.^58^ In brief, relevant atoms in the AA structure were mapped to the corresponding CG particles, and new Protein Data Bank files generated using the GROMACS editconf tool.^96^ These were then subjected to energy minimization to obtain the correct CG geometries. It has been suggested that the Martini 3.0 force field, in contrast to previous versions, can reproduce the temperature-dependent properties of compounds,^28^ and therefore, we used the same temperature series that was used by Schmidt et al.^50^ Temperatures were maintained using the canonical rescaling algorithm^97^ with a coupling time τ_T_ of 0.1 ps. Because all organic compounds considered in this work are neutral, there were no Coulomb interactions.

Then, 200 ns constant-pressure simulations were performed using Parrinello–Rahman and Berendsen barostats^53,98^ with a coupling time τ_P_ of 100 ps. To compute the melting temperature, we used the liquid/solid direct coexistence approach by conducting a series of *NpT* simulations at different temperatures as initially suggested in ref (57). For all of the simulations of organic compounds, the diffusion constant was computed for the last 5 ns of the simulations from the mean square displacement. On the basis of the temperature dependence of the diffusion constant (Figures S24–S47), the melting temperature was estimated (Table 2). It should be noted that this is rather generous definition because the structures change from the crystal state even at low temperatures (see, e.g., Figures S48 and S49).

## Data Availability

Simulations were analyzed with the GROMACS software suite.^96^ Angles were calculated using Python (NumPy and Pandas).^102,103^ Molecular images were produced using PyMOL.^104^ Matplotlib was used to generate all plots.^105^ The scripts are available from the GitHub repository.^58^

## Supporting Information Available

Supplementary figures of diffusion coefficients, lattice parameters, and RDFs (PDF)
Transparent Peer Review report available (PDF)
