# Supplementary material for: Martini on the Rocks: Can a Coarse-Grained Force Field Model Crystals?
Source: J Phys Chem Lett. 2024 Jan 23;15(4):1079–88. doi: 10.1021/acs.jpclett.4c00012 (PMC10839907; doi:10.1021/acs.jpclett.4c00012)
Supplement: Supplementary file 1 — jz4c00012_si_001.pdf [file jz4c00012_si_001.pdf]

Martini on the Rocks: Can a Coarse-Grained Force Field Model Crystals? - Supporting Information

A. Najla Hosseini and David van der Spoel*

Department of Cell and Molecular Biology, Uppsala University, Box 596, SE-75124 Uppsala, Sweden

E-mail: david.vanderspoel@icm.uu.se

Phone: +46 18 471 4205

List of Figures

S1 Deviation of lattice size of the supercell in %, over the NpT and production runs for all amyloid peptides using Parrinello-Rahman barostat (M3). S6

S2 Deviation of angles of the supercell from experimental crystal structure over the NpT and production runs for all peptides using Parrinello-Rahman barostat (M3). S7

S3 Deviation of lattice size of the supercell in %, over the NpT and production runs for all amyloid peptides using Berendsen barostat (M3). S8

S4 Deviation of angles of the supercell from experimental crystal structure over the NpT and production runs for all peptides using Berendsen barostat (M3). S9

S5 Deviation of lattice size of the supercell in %, over the NpT and production runs for all amyloid peptides considering modifications (M3′) using Parrinello-Rahman barostat. S10

S6 Deviation of angles of the supercell from experimental crystal structure over the NpT and production runs for all peptides considering modifications (M3′) using Parrinello-Rahman barostat. S11

S7 Deviation of lattice size of the supercell in %, over the NpT and production runs for all amyloid peptides considering modifications (M3′) using Berendsen barostat. S12

S8 Deviation of angles of the supercell from experimental crystal structure over the NpT and production runs for all peptides considering modifications (M3′) using Berendsen barostat. S13

S9 Deviation of lattice size of the supercell in %, over the NpT and production runs for all amyloid peptides considering modifications (M3″) using Parrinello-Rahman barostat. S14

S10 Deviation of angles of the supercell from experimental crystal structure over the NpT and production runs for all peptides considering modifications (M3″) using Parrinello-Rahman barostat. S15
S11 Deviation of lattice size of the supercell in %, over the NpT and production runs for all amyloid peptides considering modifications (M3″) using Berendsen barostat. S16
S12 Deviation of angles of the supercell from experimental crystal structure over the NpT and production runs for all peptides considering modifications (M3″) using Berendsen barostat. S17
S13 Deviation of lattice size of the NNFGAIL in %, over the NpT and production runs using reaction-field and particle mesh Ewald (PME) for the (M3″) force field using Berendsen barostat at 293 K. S18
S14 Deviation of lattice size of the NNFGAIL in %, over the NpT and production runs using reaction-field and PME for the (M3″) force field using Berendsen barostat at 100 K. S18
S15 Deviation of angles of the NNFGAIL from experimental crystal structure over the NpT and production runs using reaction-field and PME considering (M3″) force field model with Berendsen barostat at 293 K. S19
S16 Deviation of angles of the NNFGAIL from experimental crystal structure over the NpT and production runs using reaction-field and PME considering (M3″) force field model with Berendsen barostat at 100 K. S19
S17 Radial distribution function (RDF) of the backbone of NNQQ1 at cryo and room temperatures for both initial and simulation (M3) using the Parrinello barostat. S20
S18 RDF of the backbone of NNQQ1 at cryo and room temperatures for both initial and simulation (M3) using the Berendsen barostat. S20

S19 RDF of the backbone of NNQQ1 at cryo and room temperatures for both initial and simulation using the Parrinello barostat (M3′). S21
S20 RDF of the backbone of NNQQ1 at cryo and room temperatures for both initial and simulation using the Berendsen barostat (M3′). S21
S21 RDF of the backbone of NNQQ1 at cryo and room temperatures for both initial and simulation using the Parrinello barostat (M3″). S22
S22 RDF of the backbone of NNQQ1 at cryo and room temperatures for both initial and simulation using the Berendsen barostat (M3″). S22
S23 RDF of the backbone of NNQQ1 at cryo and room temperatures for both experiment and simulation using the Berendsen barostat with CHARMM36m. S23
S24 Diffusion as a function of temperature using Parrinello-Rahman barostat. . . S23
S25 Diffusion as a function of temperature using Berendsen barostat. S24
S26 Diffusion as a function of temperature using Parrinello-Rahman barostat. . . S24
S27 Diffusion as a function of temperature using Berendsen barostat. S25
S28 Diffusion as a function of temperature. S25
S29 Diffusion as a function of temperature. S26
S30 Diffusion as a function of temperature. S26
S31 Diffusion as a function of temperature. S27
S32 Diffusion as a function of temperature. S27
S33 Diffusion as a function of temperature. S28
S34 Diffusion as a function of temperature. S28
S35 Diffusion as a function of temperature. S29
S36 Diffusion as a function of temperature. S29
S37 Diffusion as a function of temperature. S30
S38 Diffusion as a function of temperature. S30
S39 Diffusion as a function of temperature. S31
S40 Diffusion as a function of temperature. S31

S41 Diffusion as a function of temperature. S31
S42 Diffusion as a function of temperature. S32
S43 Diffusion as a function of temperature. S32
S44 Diffusion as a function of temperature. S32
S45 Diffusion as a function of temperature. S33
S46 Diffusion as a function of temperature. S33
S47 Diffusion as a function of temperature. S33
S48 Radial distribution function (RDF) of pyridine at 5 K for both experiment and simulation using the Berendsen barostat. S34
S49 Radial distribution function (RDF) of phenol at 5 K for both experiment and simulation using the Berendsen barostat. S35
S50 Correlation between experimental melting temperature and simulated melting temperatures for 12 organic compounds (see table 2 in main text). S35

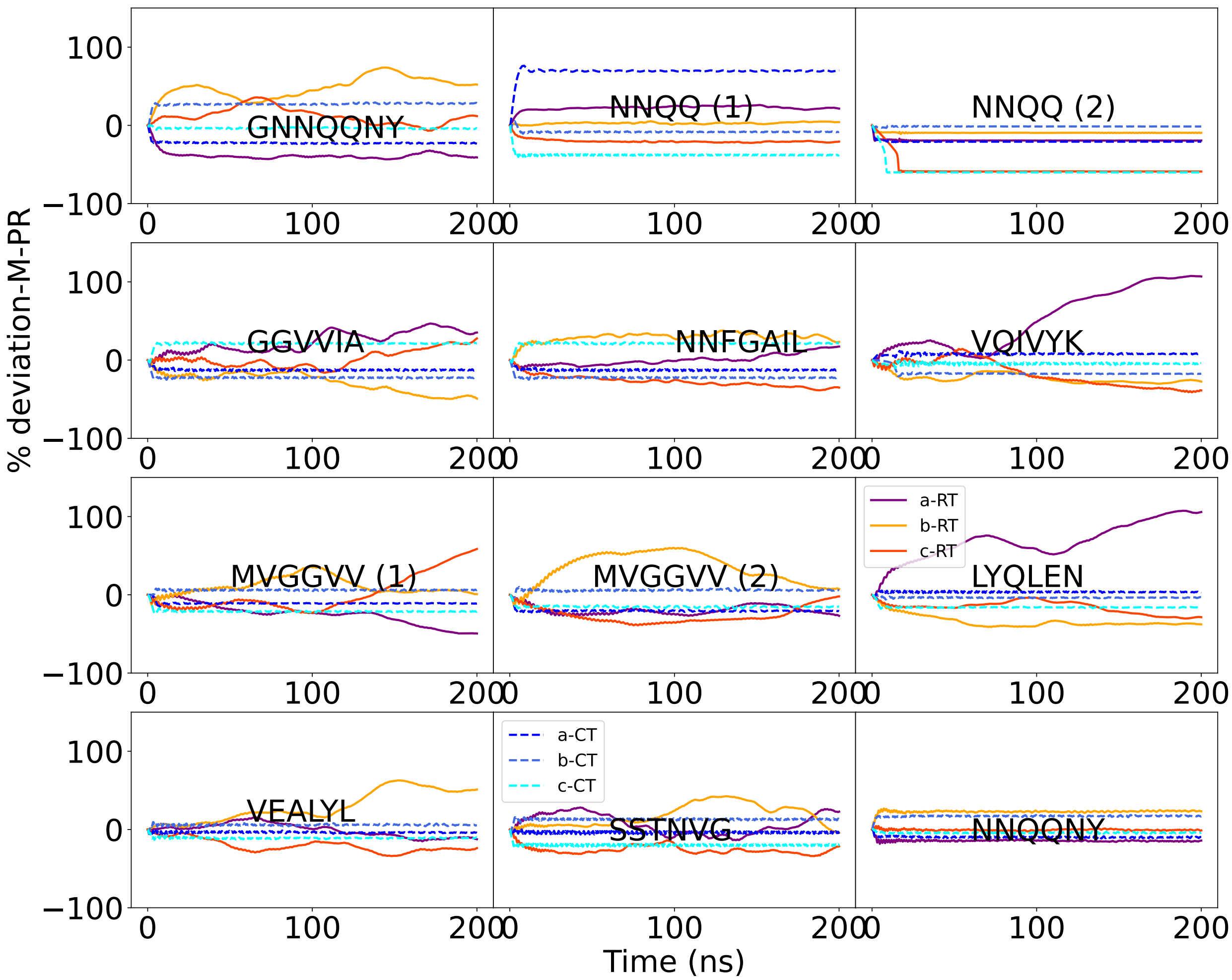

Figure S1: Deviation of lattice size of the supercell in %, over the NpT and production runs for all amyloid peptides using Parrinello-Rahman barostat (M3).

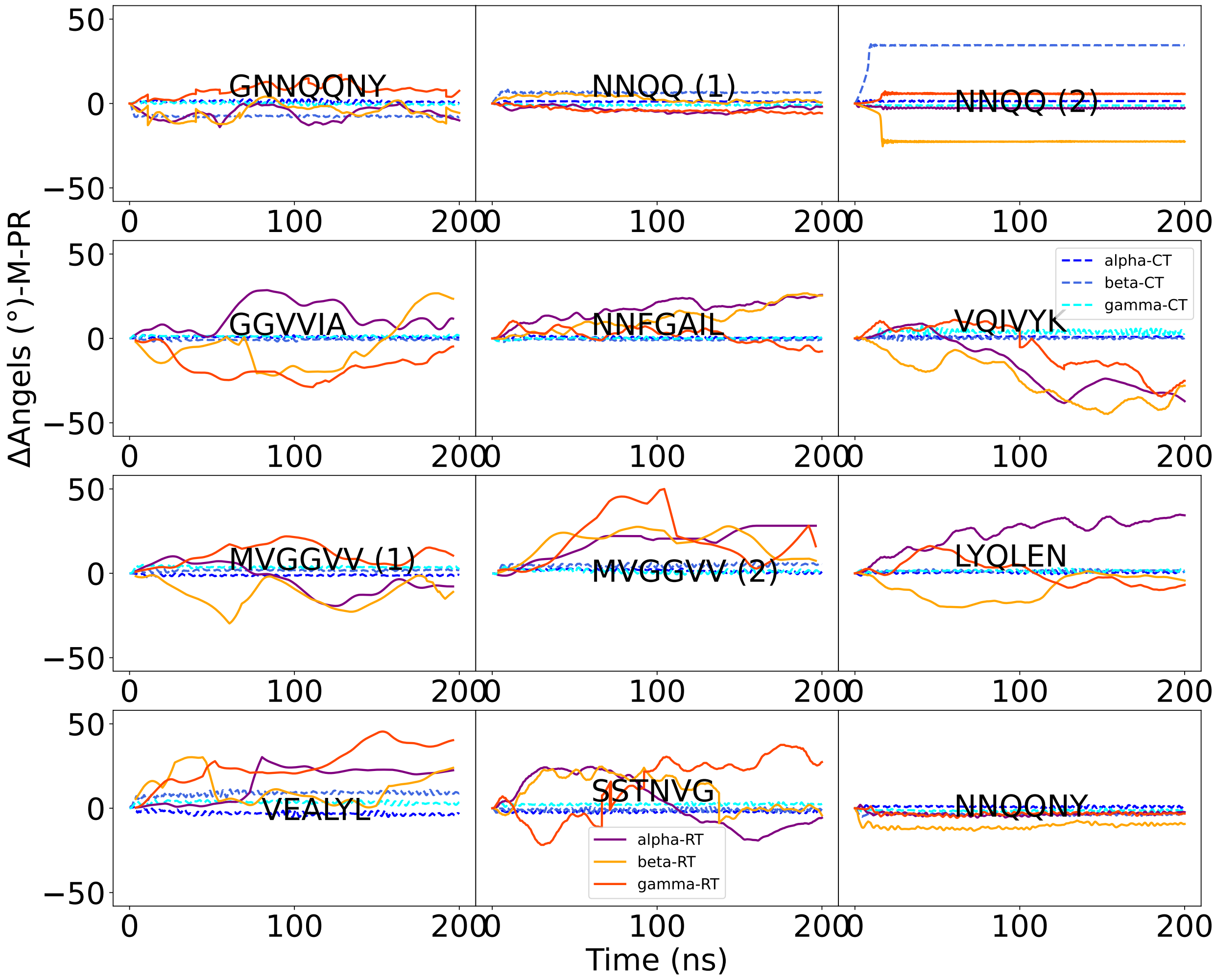

Figure S2: Deviation of angles of the supercell from experimental crystal structure over the NpT and production runs for all peptides using Parrinello-Rahman barostat (M3).

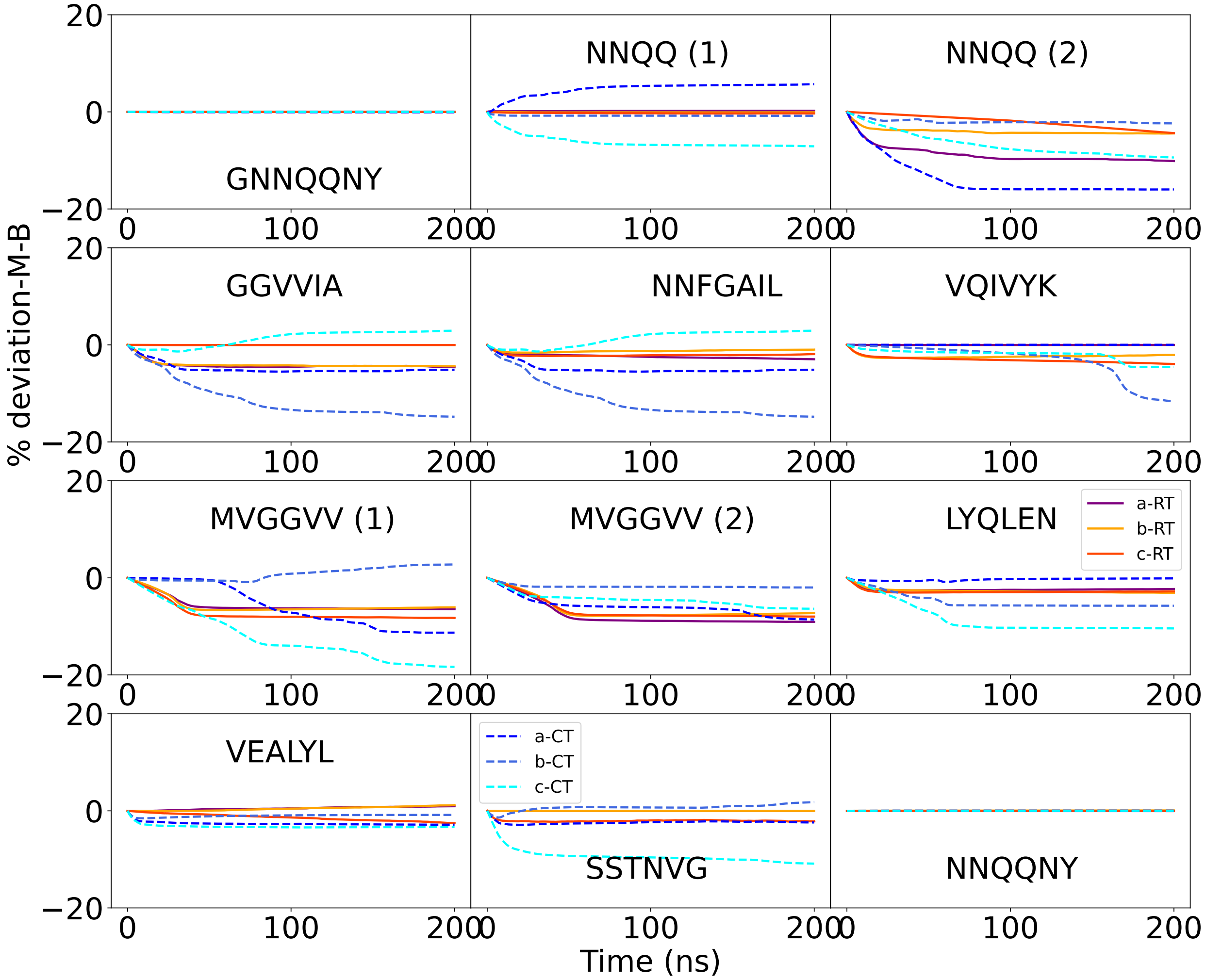

Figure S3: Deviation of lattice size of the supercell in %, over the NpT and production runs for all amyloid peptides using Berendsen barostat (M3).

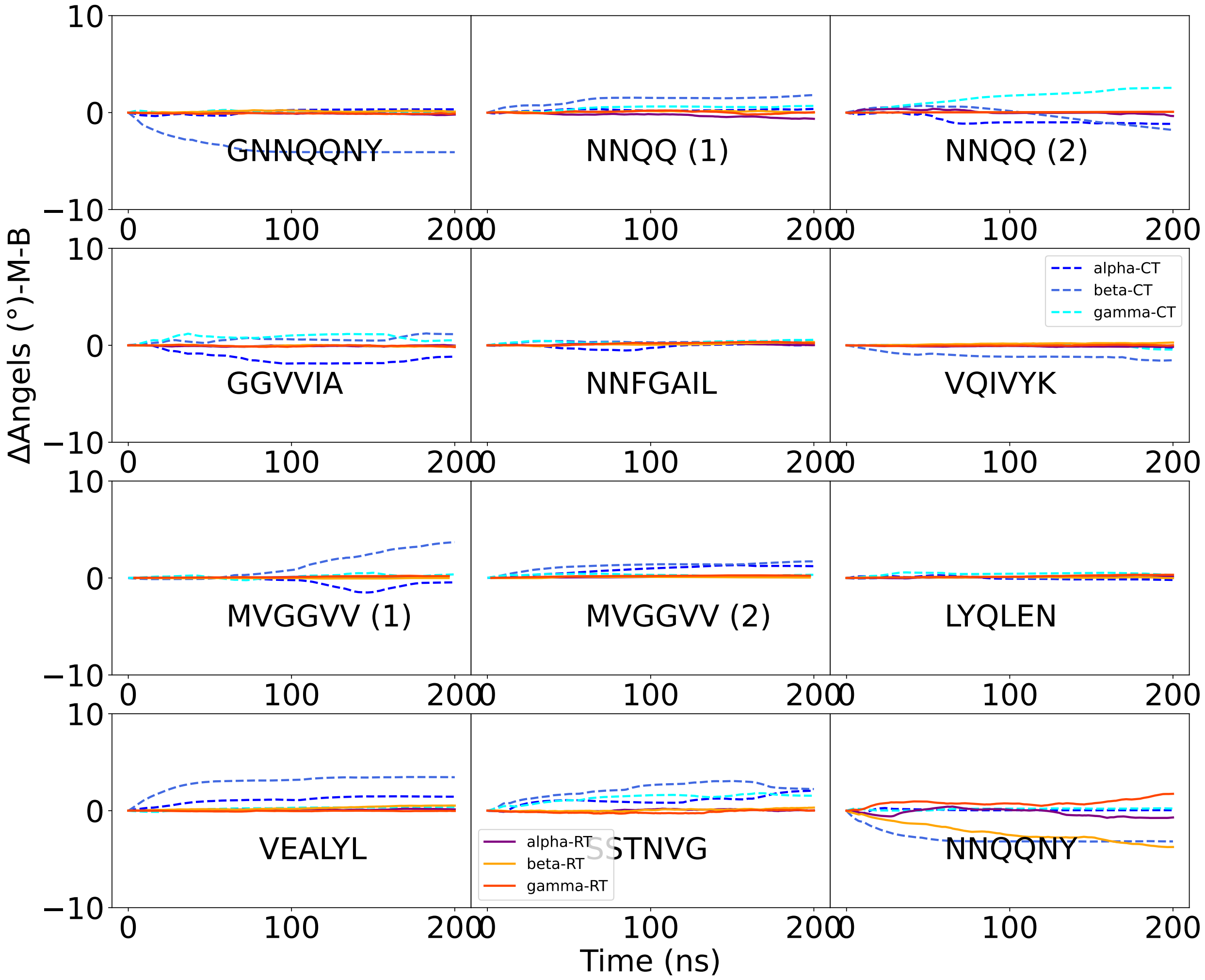

Figure S4: Deviation of angles of the supercell from experimental crystal structure over the NpT and production runs for all peptides using Berendsen barostat (M3).

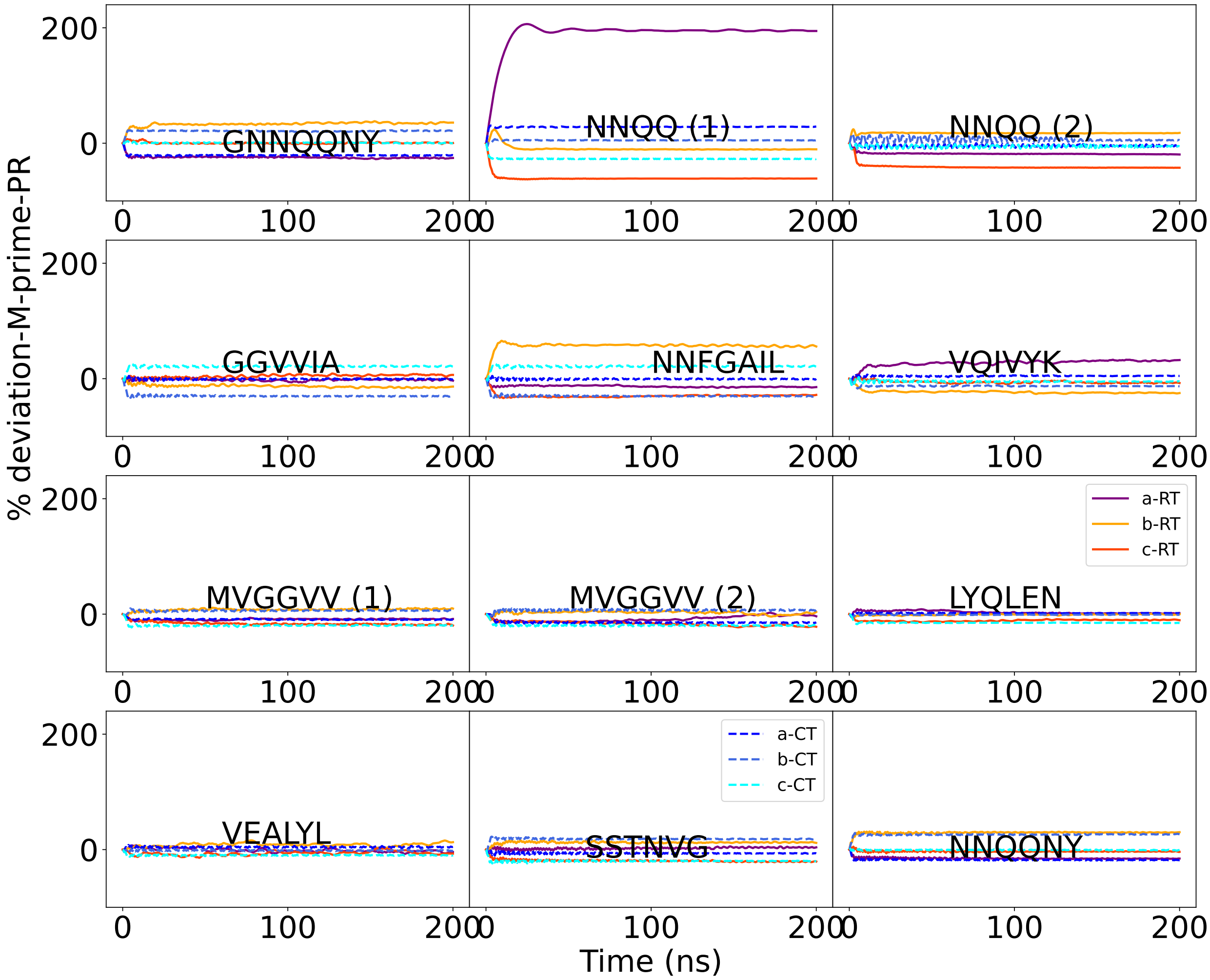

Figure S5: Deviation of lattice size of the supercell in %, over the NpT and production runs for all amyloid peptides considering modifications (M3′) using Parrinello-Rahman barostat.

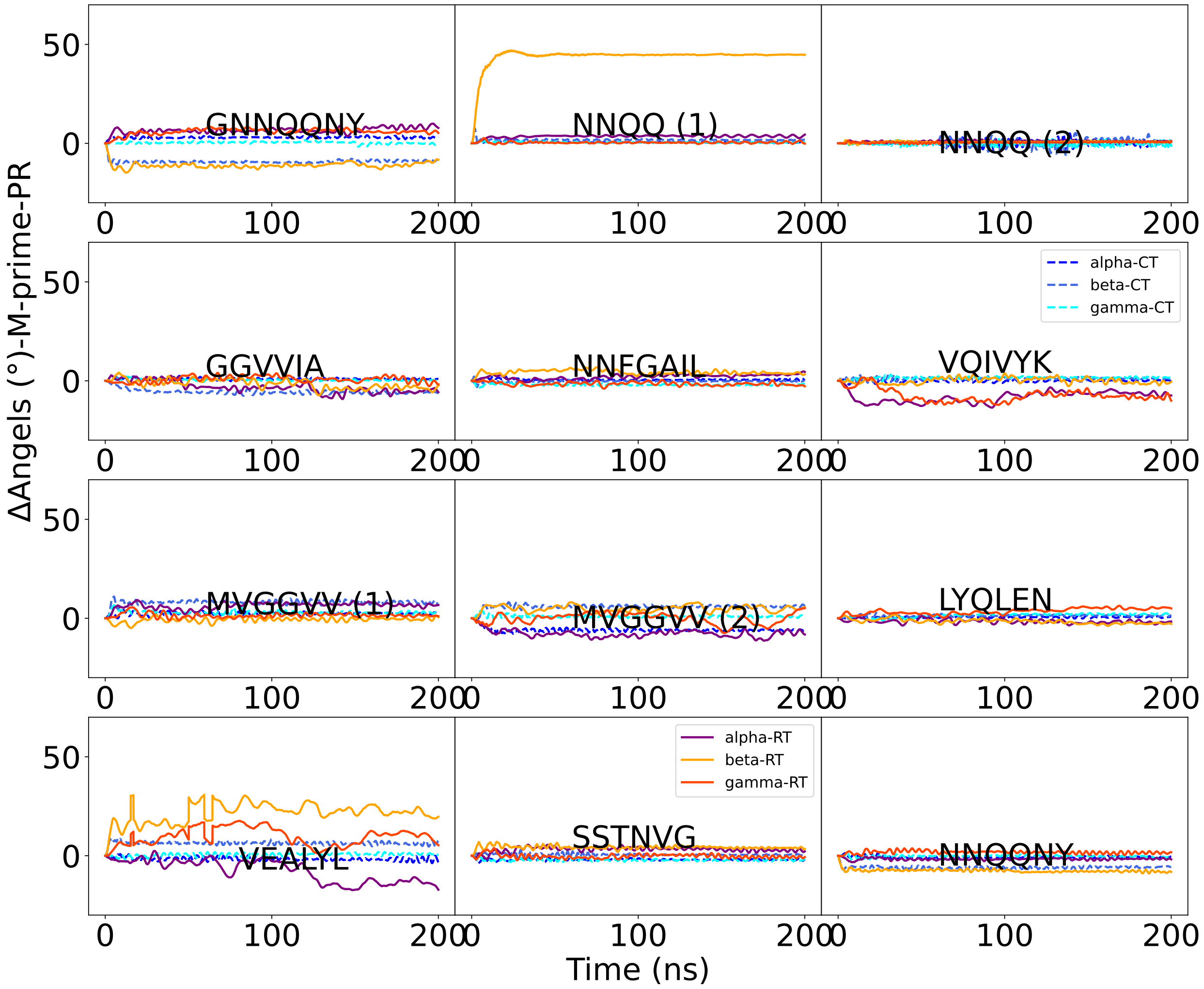

Figure S6: Deviation of angles of the supercell from experimental crystal structure over the NpT and production runs for all peptides considering modifications (M3′) using Parrinello-Rahman barostat.

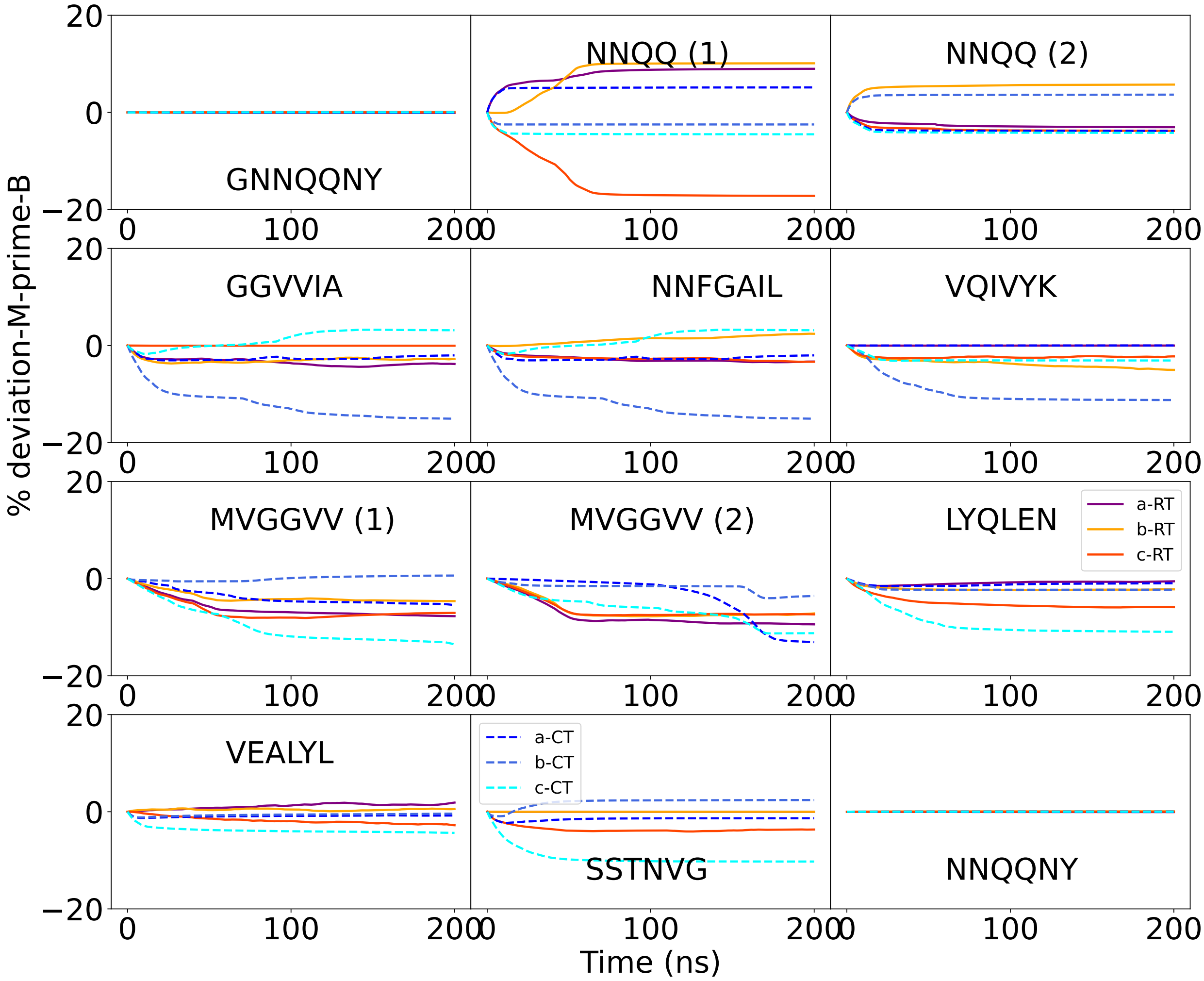

Figure S7: Deviation of lattice size of the supercell in %, over the NpT and production runs for all amyloid peptides considering modifications (M3′) using Berendsen barostat.

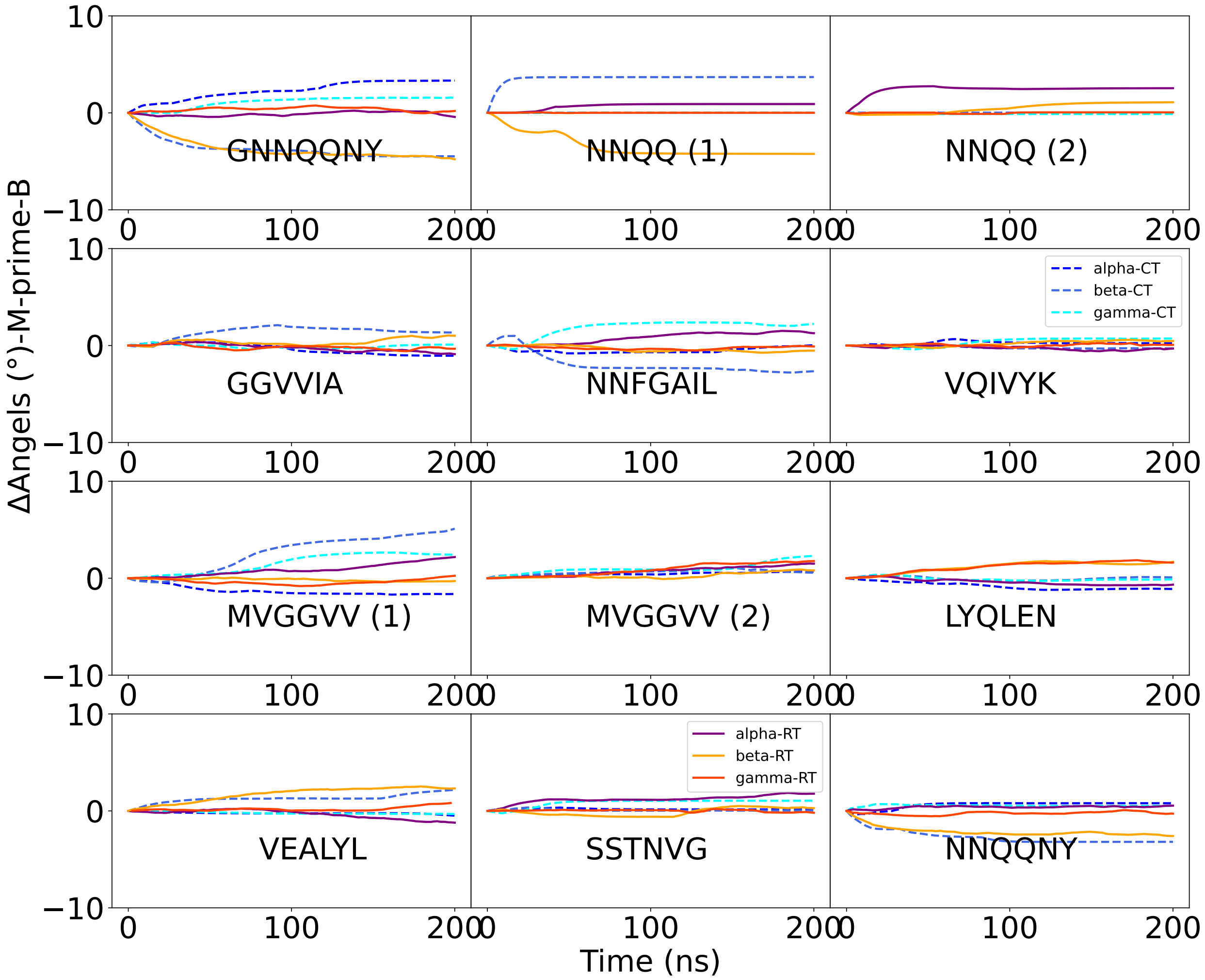

Figure S8: Deviation of angles of the supercell from experimental crystal structure over the NpT and production runs for all peptides considering modifications (M3′) using Berendsen barostat.

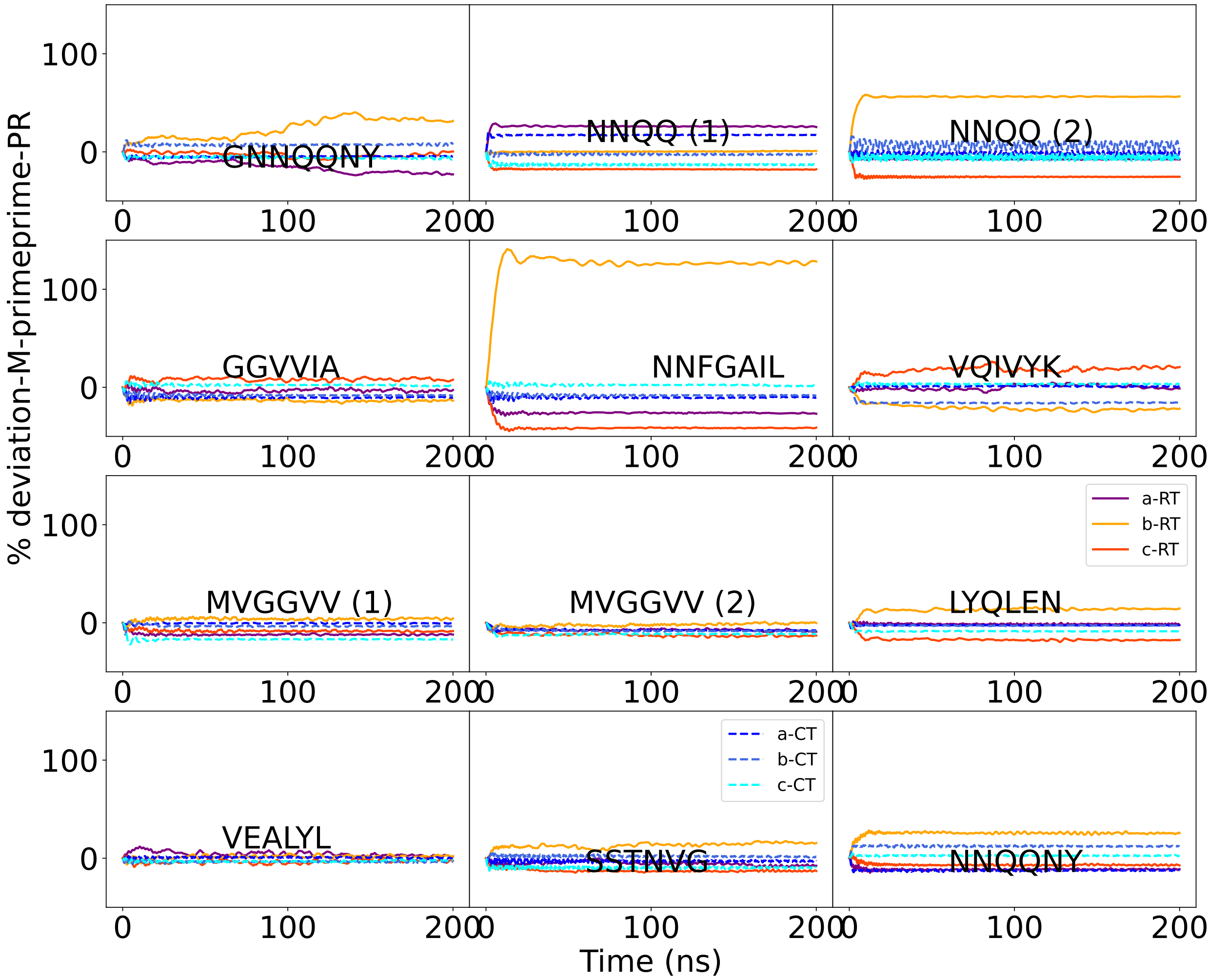

Figure S9: Deviation of lattice size of the supercell in %, over the NpT and production runs for all amyloid peptides considering modifications (M3″) using Parrinello-Rahman barostat.

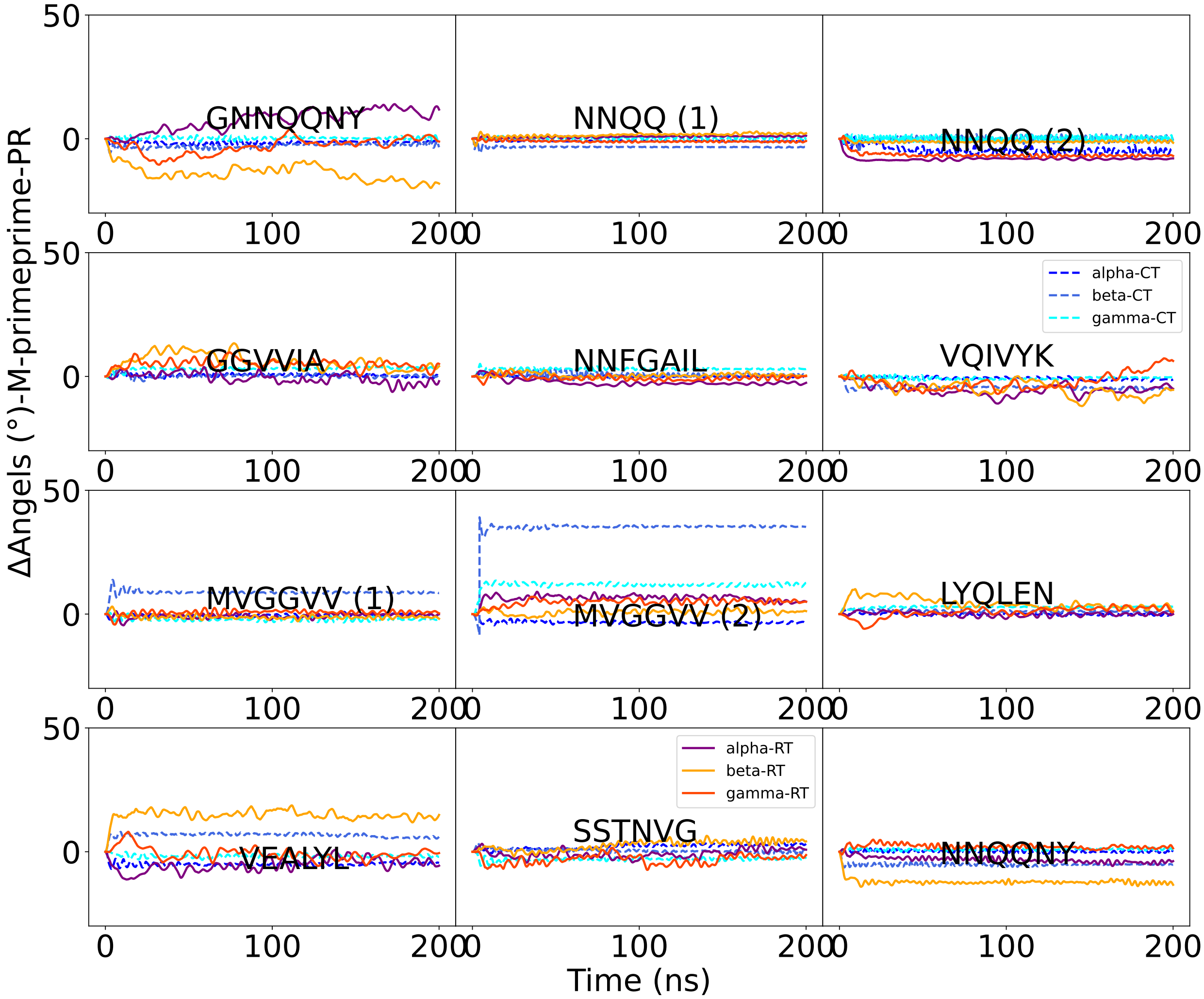

Figure S10: Deviation of angles of the supercell from experimental crystal structure over the NpT and production runs for all peptides considering modifications (M3″) using Parrinello-Rahman barostat.

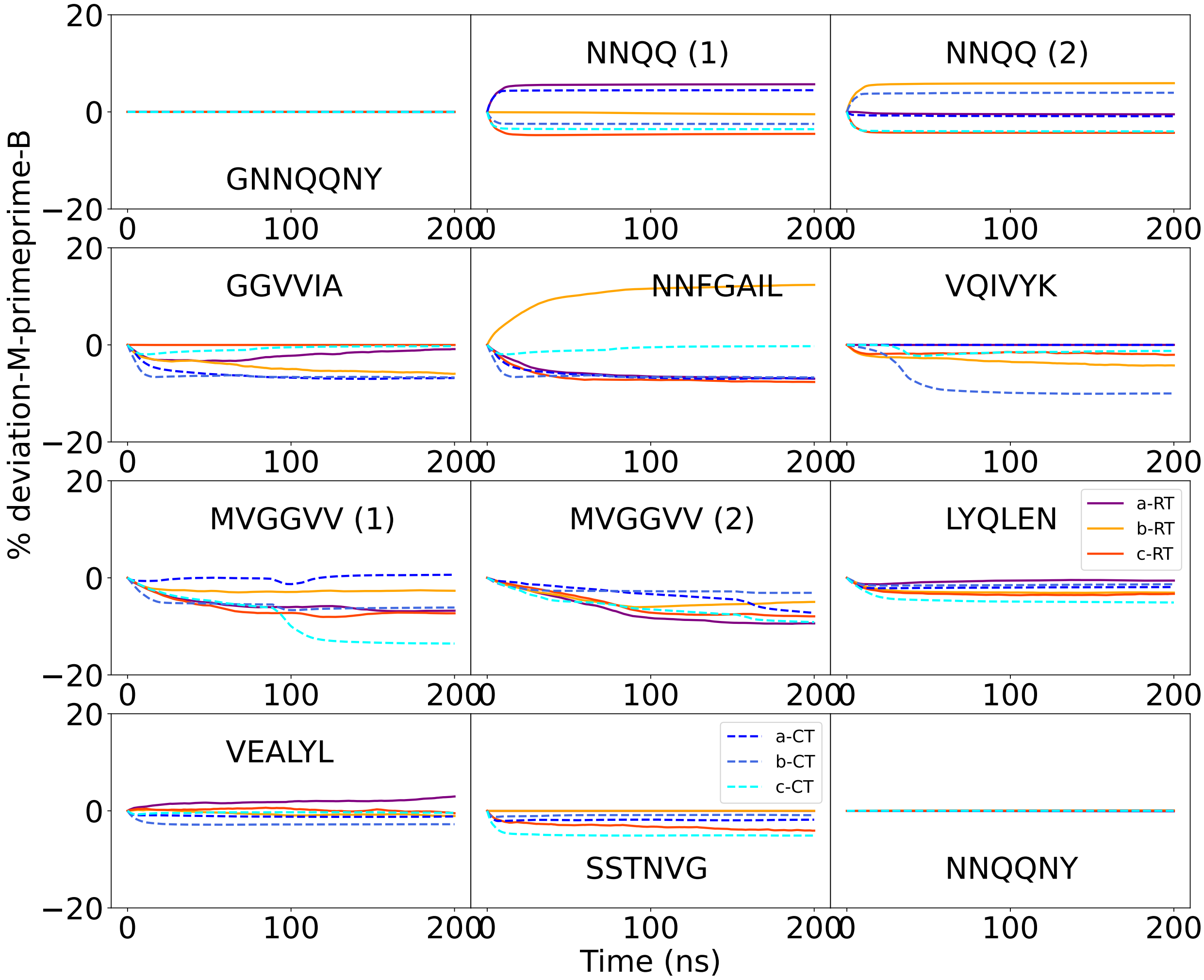

Figure S11: Deviation of lattice size of the supercell in %, over the NpT and production runs for all amyloid peptides considering modifications (M3″) using Berendsen barostat.

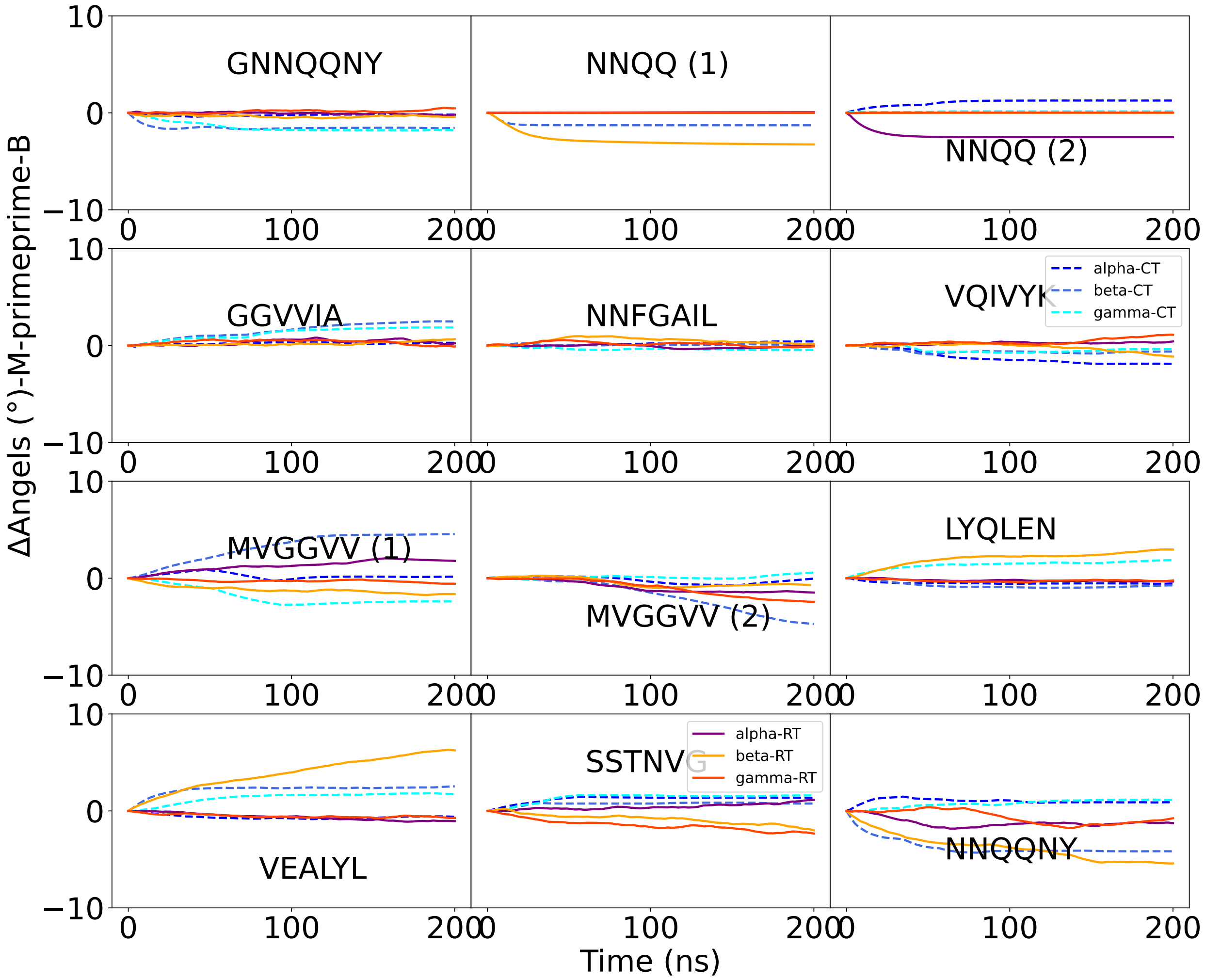

Figure S12: Deviation of angles of the supercell from experimental crystal structure over the NpT and production runs for all peptides considering modifications (M3″) using Berendsen barostat.

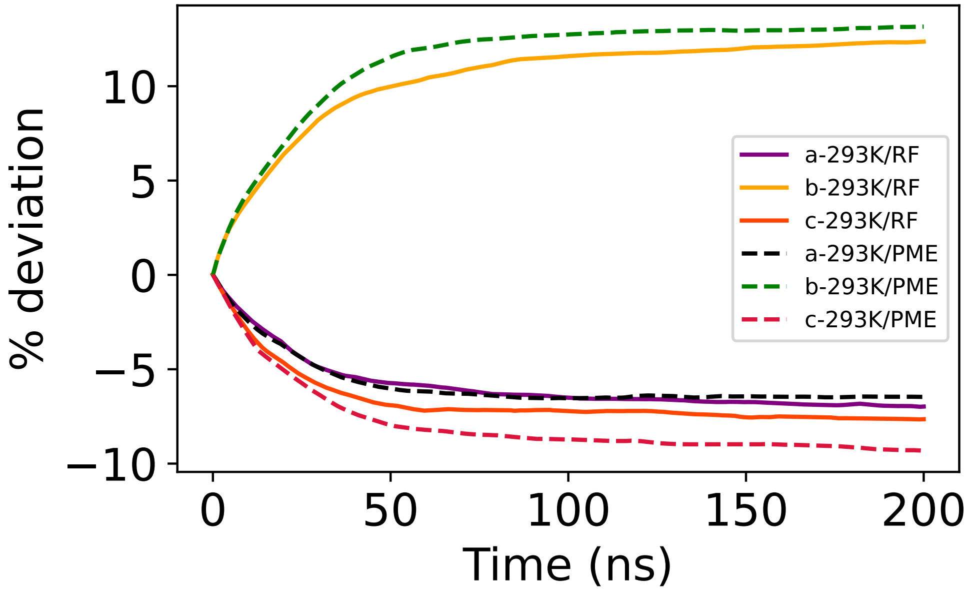

Figure S13: Deviation of lattice size of the NNFGAIL in %, over the NpT and production runs using reaction-field and particle mesh Ewald (PME) for the (M3″) force field using Berendsen barostat at 293 K.

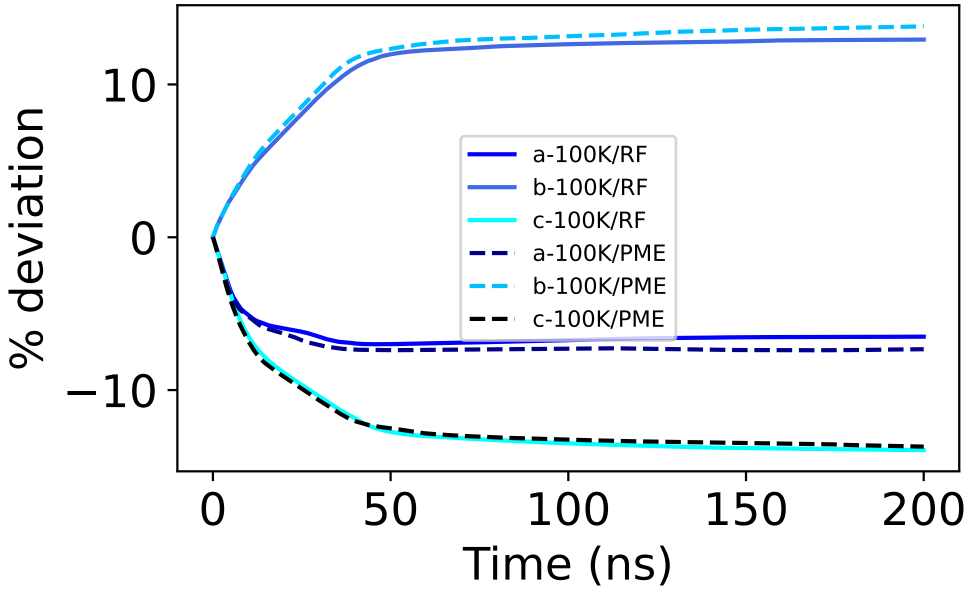

Figure S14: Deviation of lattice size of the NNFGAIL in %, over the NpT and production runs using reaction-field and PME for the (M3″) force field using Berendsen barostat at 100 K.

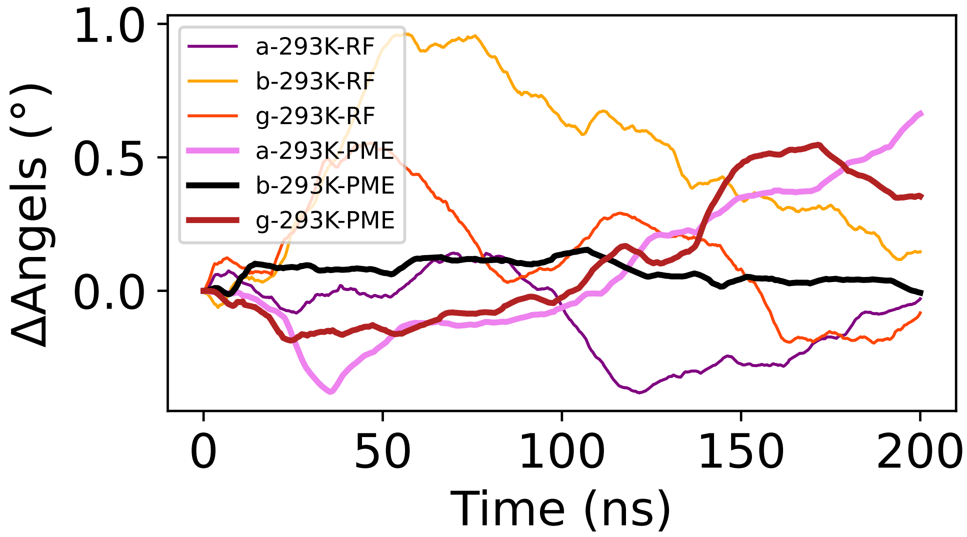

Figure S15: Deviation of angles of the NNFGAIL from experimental crystal structure over the NpT and production runs using reaction-field and PME considering (M3″) force field model with Berendsen barostat at 293 K.

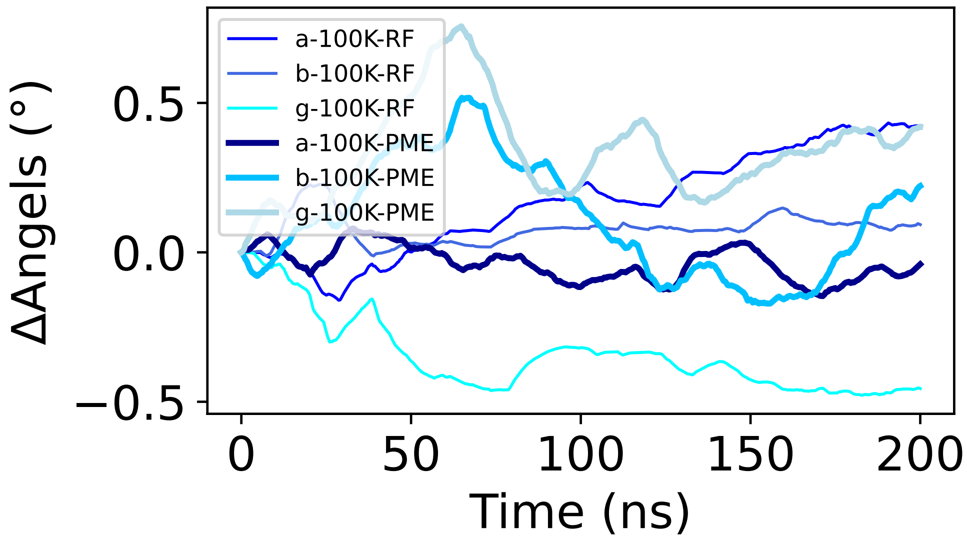

Figure S16: Deviation of angles of the NNFGAIL from experimental crystal structure over the NpT and production runs using reaction-field and PME considering (M3″) force field model with Berendsen barostat at 100 K.

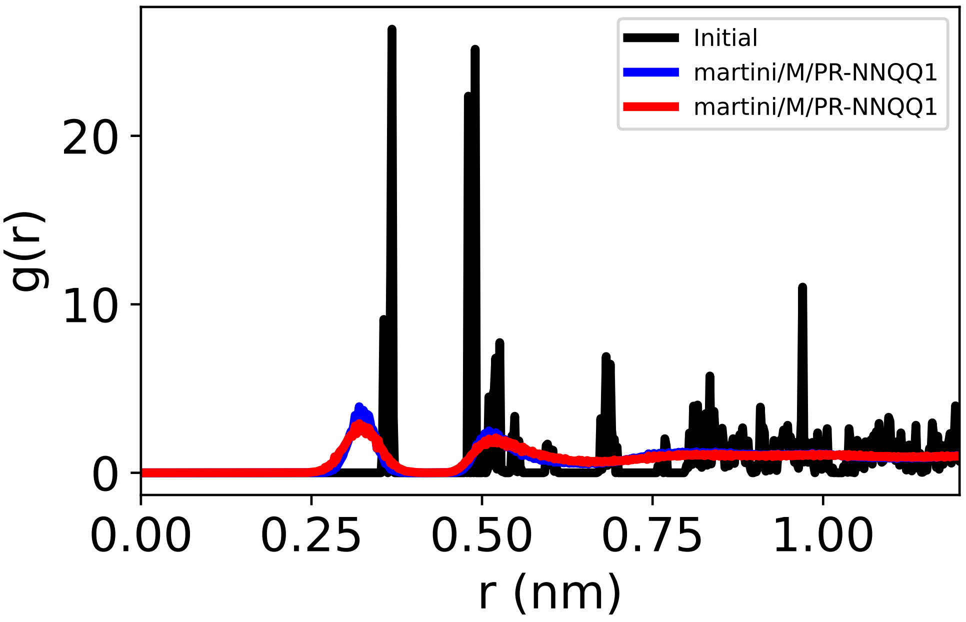

Figure S17: Radial distribution function (RDF) of the backbone of NNQQ1 at cryo and room temperatures for both initial and simulation (M3) using the Parrinello barostat.

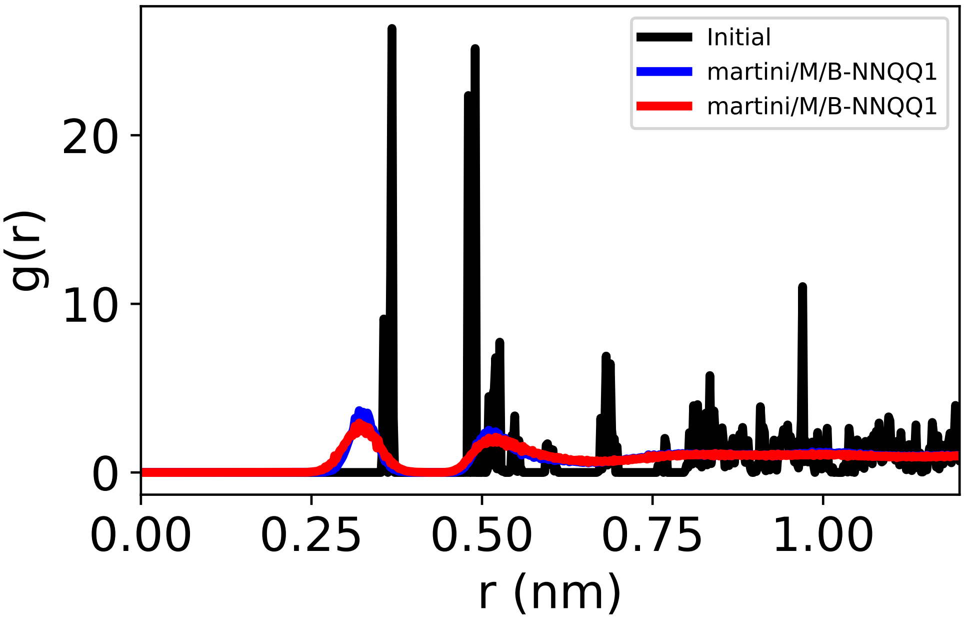

Figure S18: RDF of the backbone of NNQQ1 at cryo and room temperatures for both initial and simulation (M3) using the Berendsen barostat.

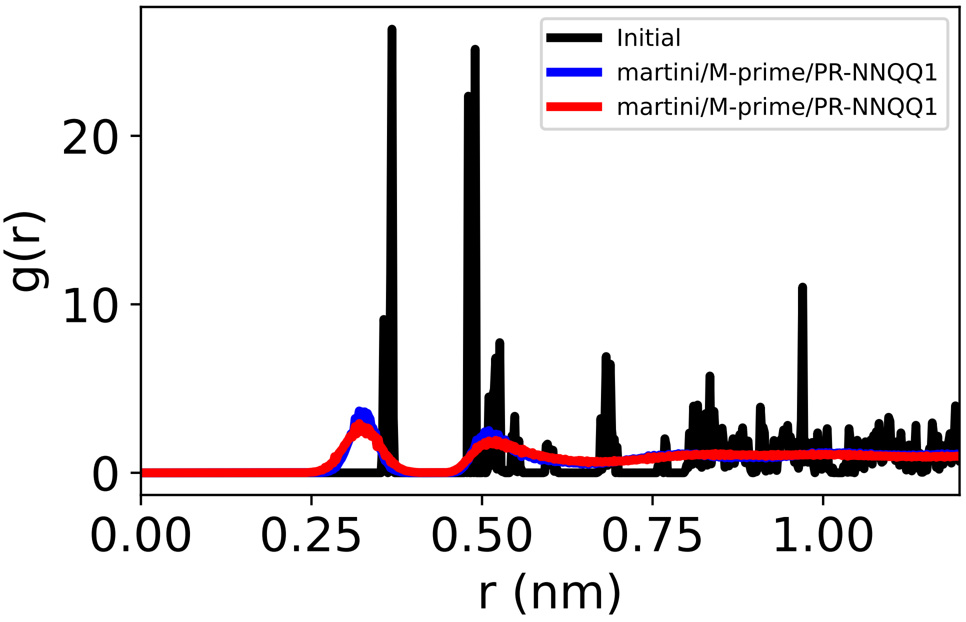

Figure S19: RDF of the backbone of NNQQ1 at cryo and room temperatures for both initial and simulation using the Parrinello barostat (M3′).

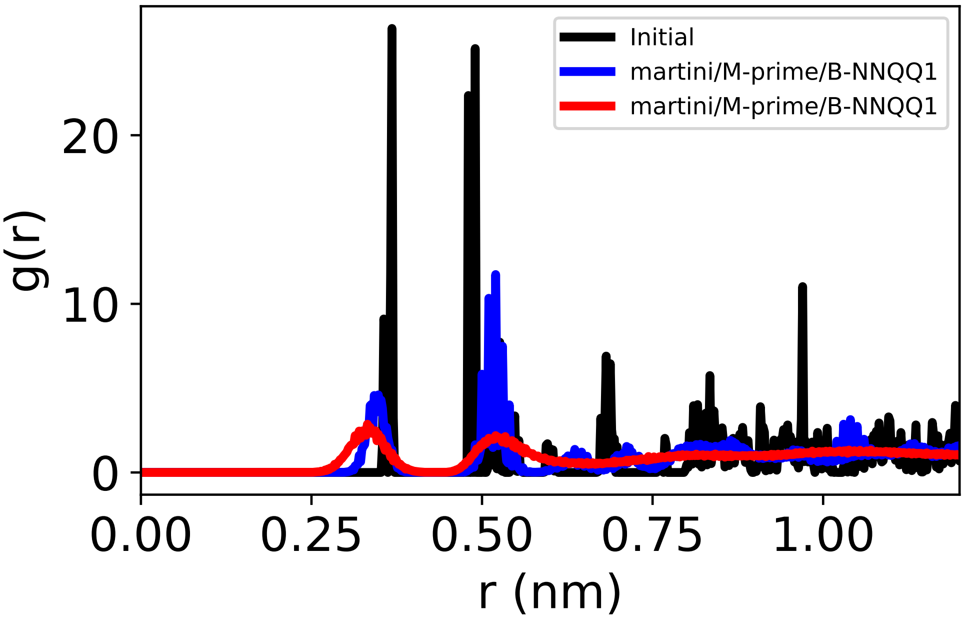

Figure S20: RDF of the backbone of NNQQ1 at cryo and room temperatures for both initial and simulation using the Berendsen barostat (M3′).

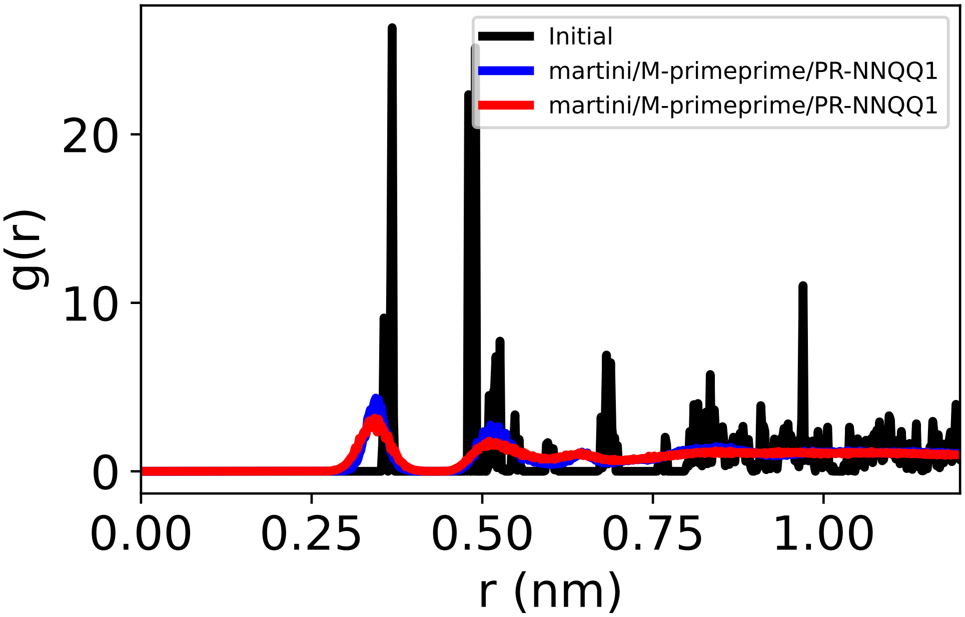

Figure S21: RDF of the backbone of NNQQ1 at cryo and room temperatures for both initial and simulation using the Parrinello barostat (M3″).

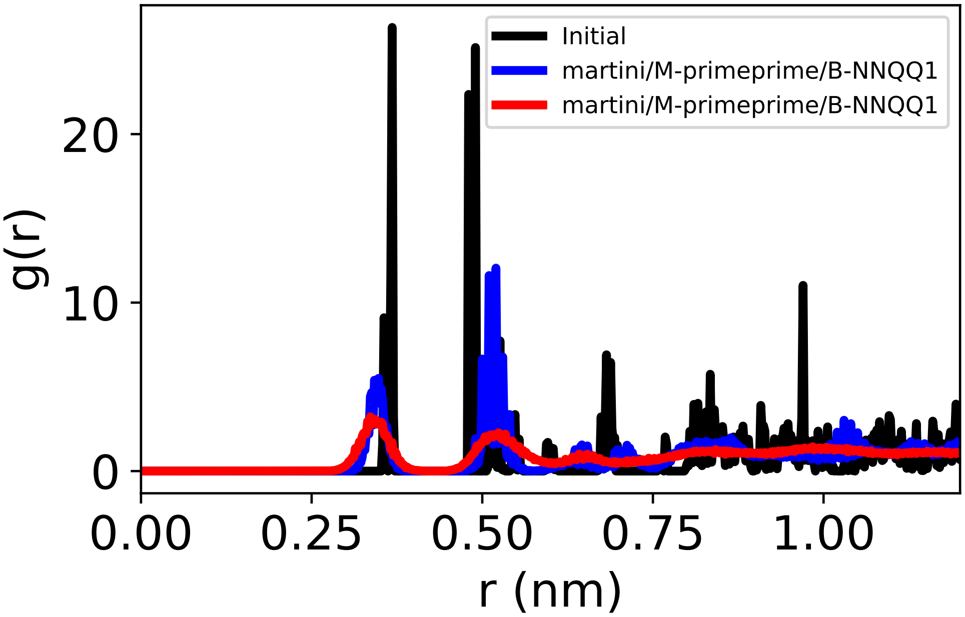

Figure S22: RDF of the backbone of NNQQ1 at cryo and room temperatures for both initial and simulation using the Berendsen barostat (M3″).

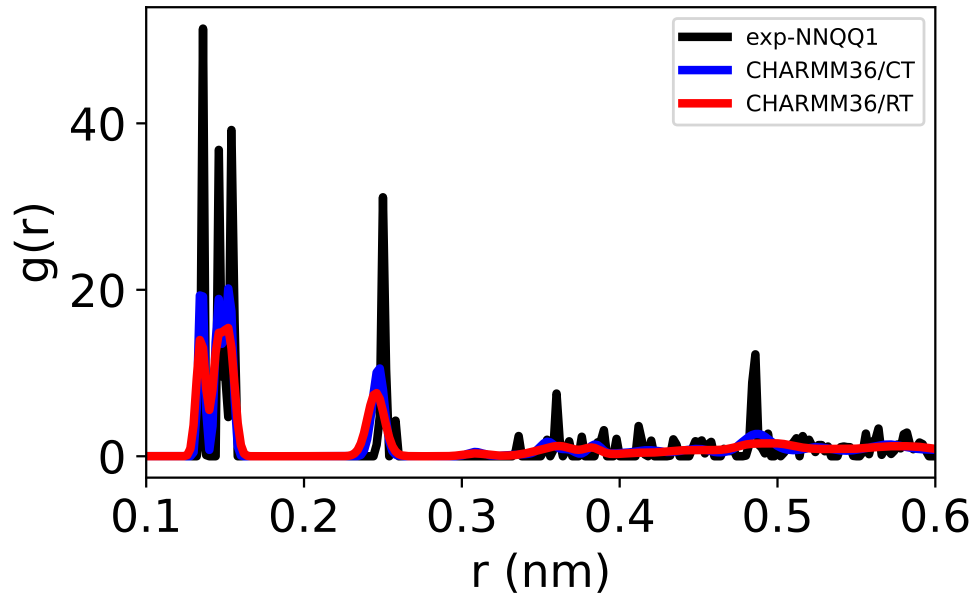

Figure S23: RDF of the backbone of NNQQ1 at cryo and room temperatures for both experiment and simulation using the Berendsen barostat with CHARMM36m.

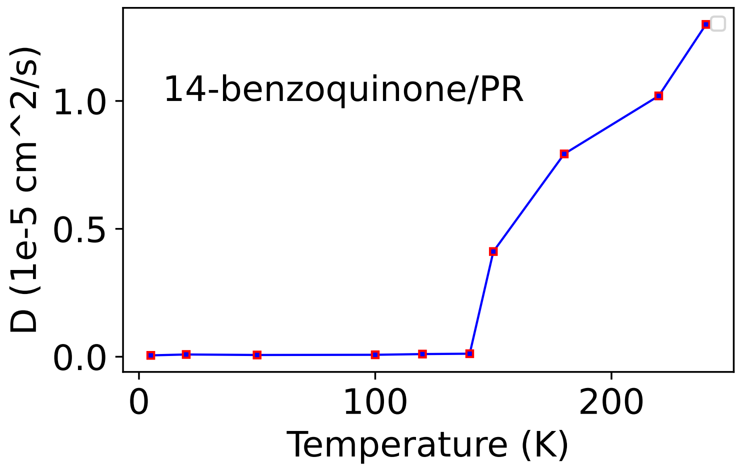

Figure S24: Diffusion as a function of temperature using Parrinello-Rahman barostat.

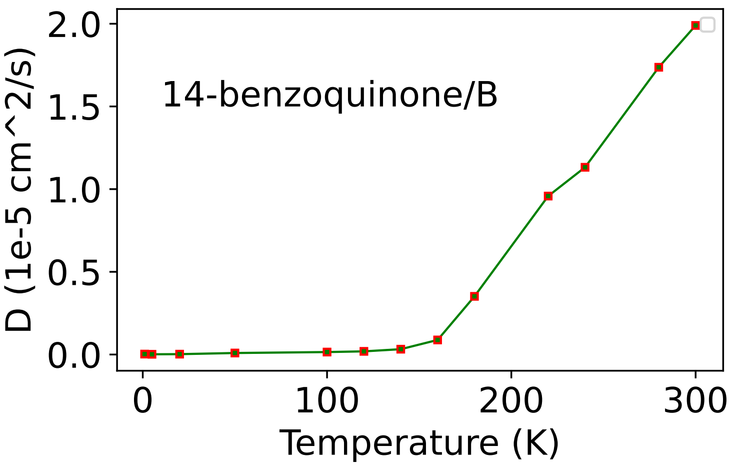

Figure S25: Diffusion as a function of temperature using Berendsen barostat.

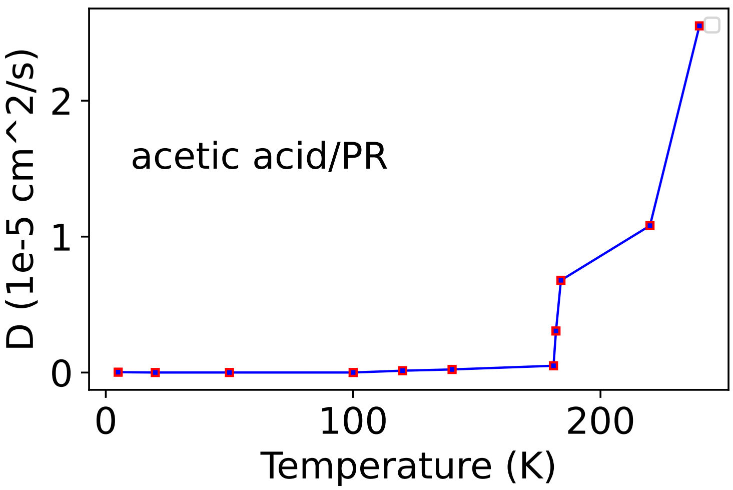

Figure S26: Diffusion as a function of temperature using Parrinello-Rahman barostat.

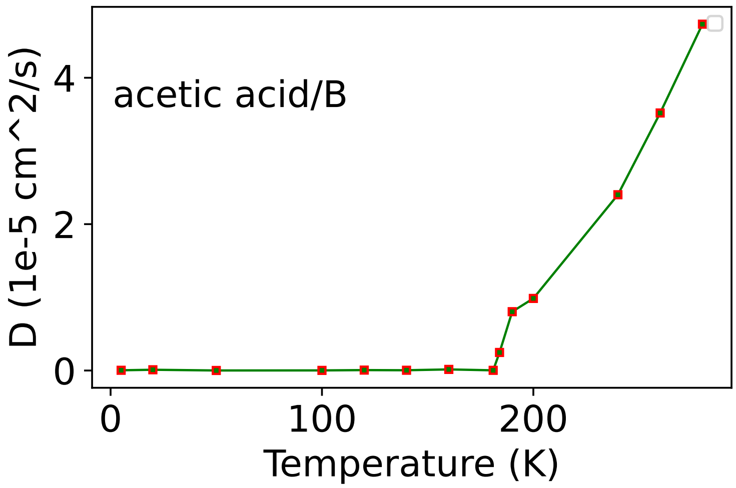

Figure S27: Diffusion as a function of temperature using Berendsen barostat.

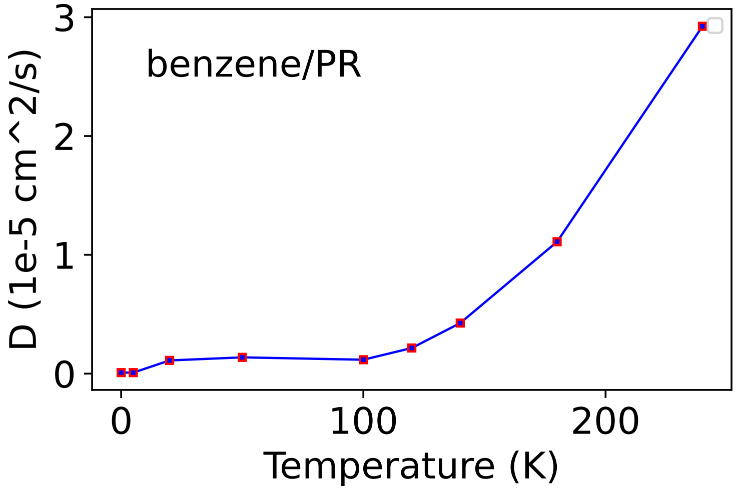

Figure S28: Diffusion as a function of temperature.

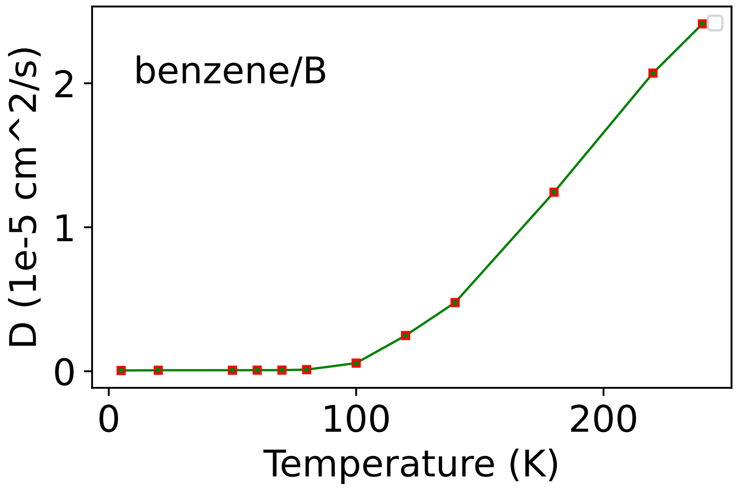

Figure S29: Diffusion as a function of temperature.

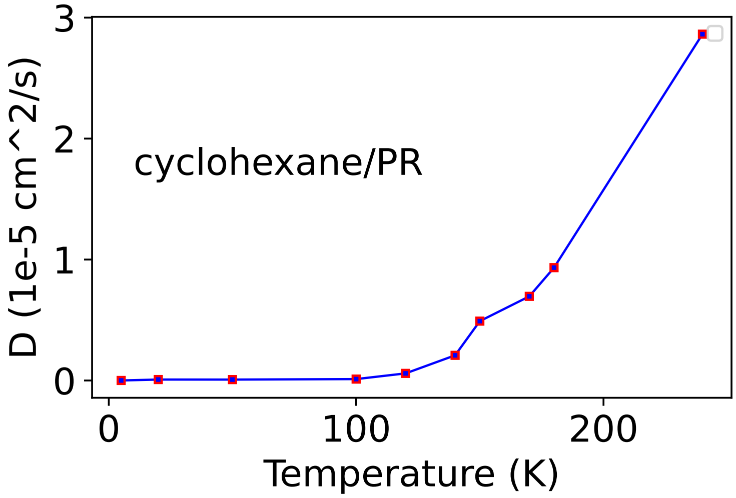

Figure S30: Diffusion as a function of temperature.

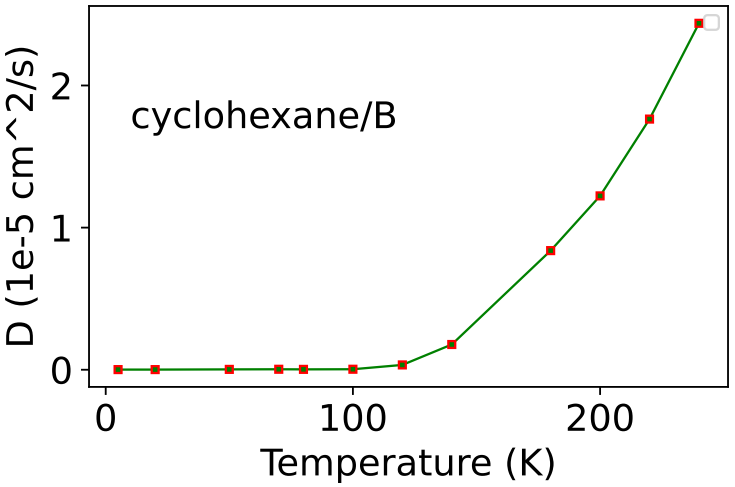

Figure S31: Diffusion as a function of temperature.

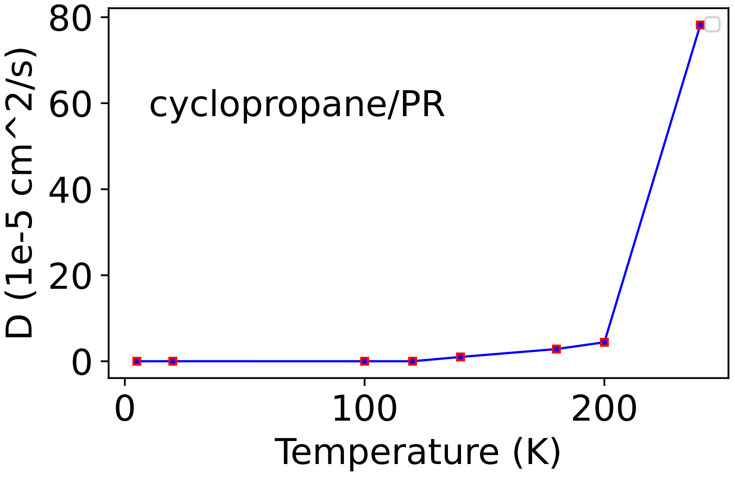

Figure S32: Diffusion as a function of temperature.

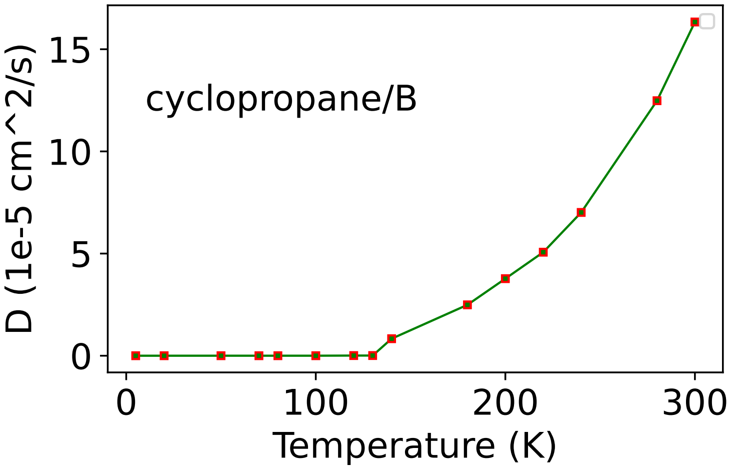

Figure S33: Diffusion as a function of temperature.

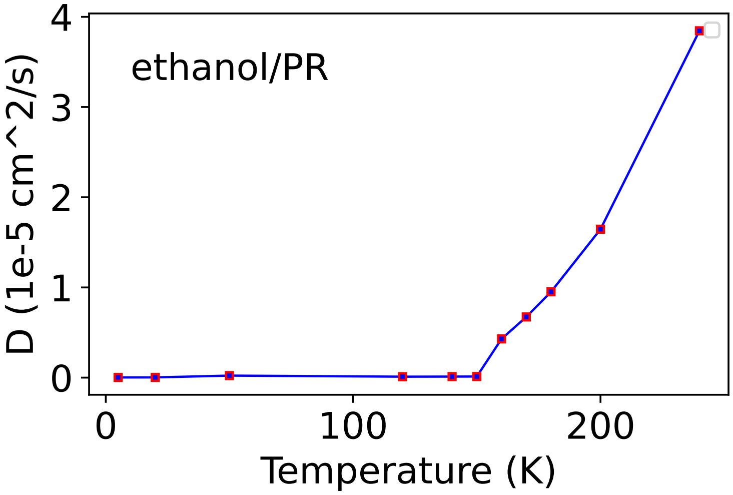

Figure S34: Diffusion as a function of temperature.

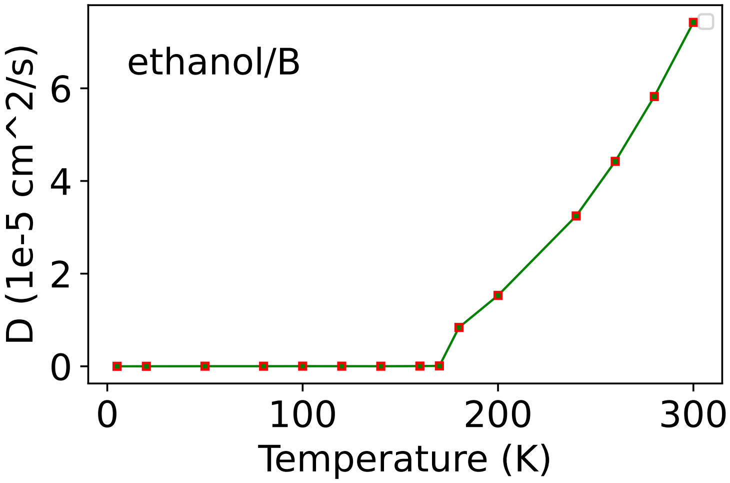

Figure S35: Diffusion as a function of temperature.

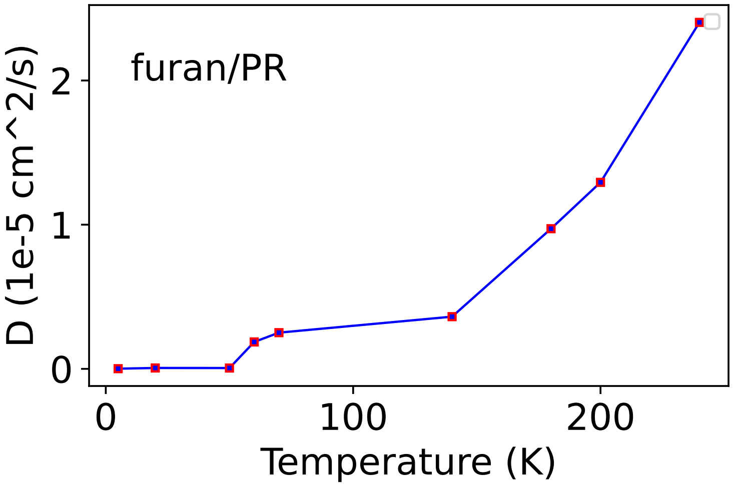

Figure S36: Diffusion as a function of temperature.

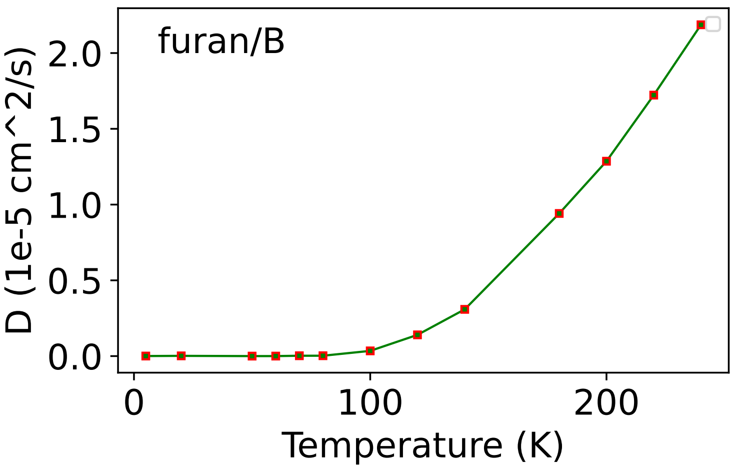

Figure S37: Diffusion as a function of temperature.

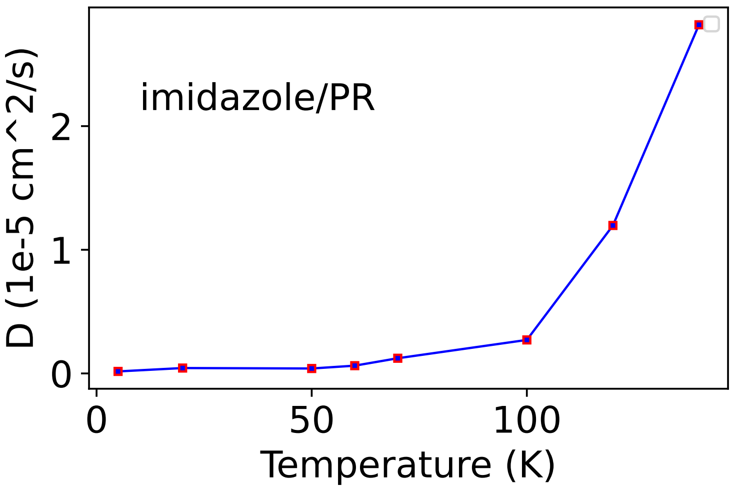

Figure S38: Diffusion as a function of temperature.

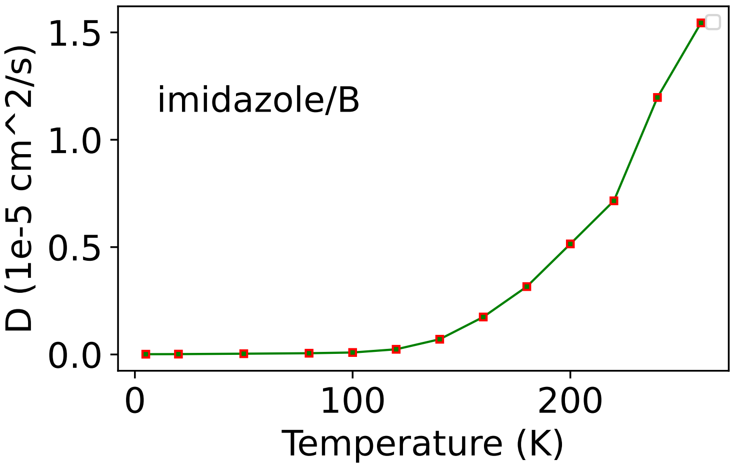

Figure S39: Diffusion as a function of temperature.

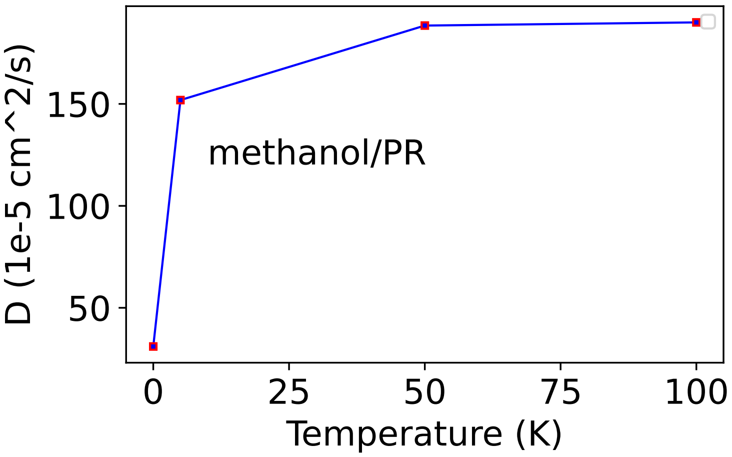

Figure S40: Diffusion as a function of temperature.

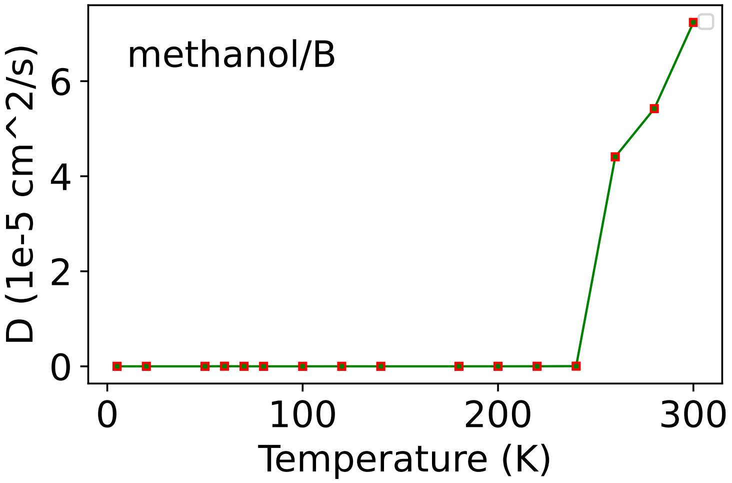

Figure S41: Diffusion as a function of temperature.

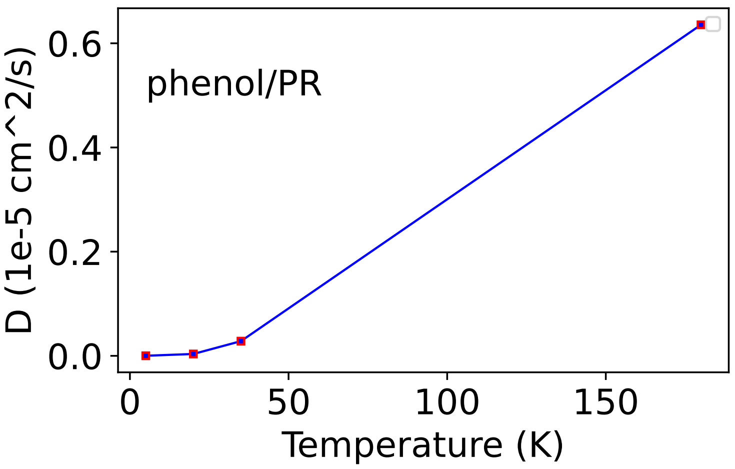

Figure S42: Diffusion as a function of temperature.

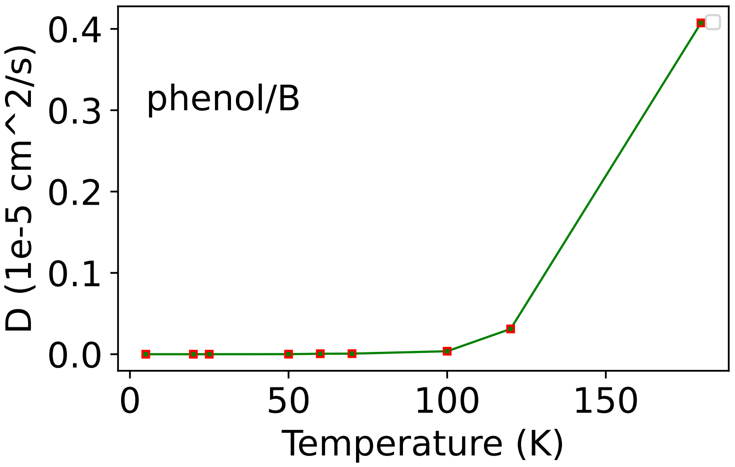

Figure S43: Diffusion as a function of temperature.

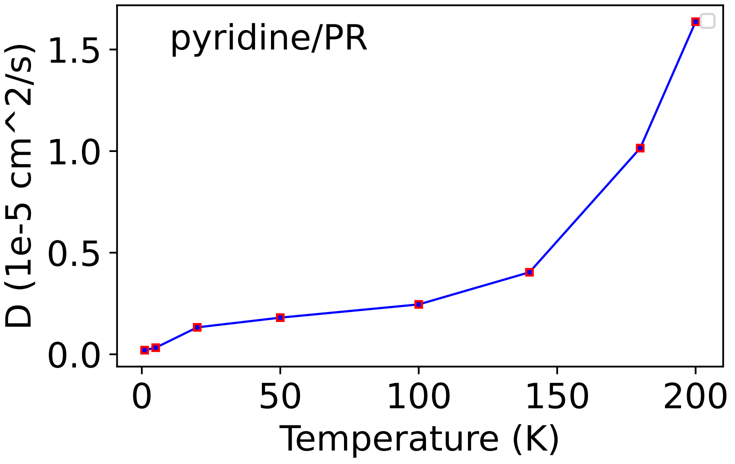

Figure S44: Diffusion as a function of temperature.

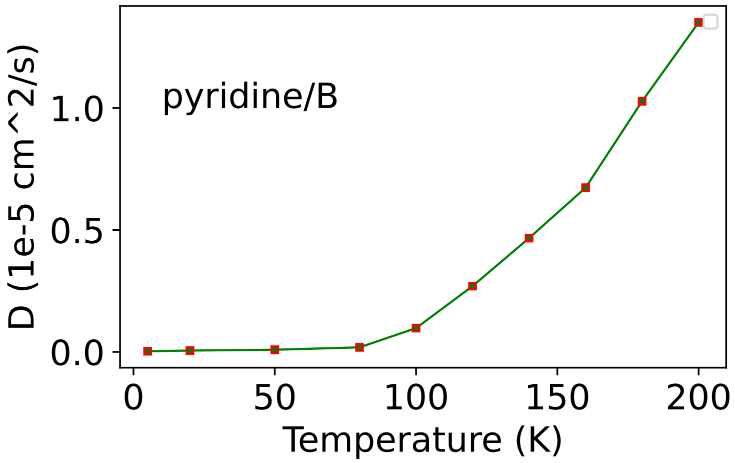

Figure S45: Diffusion as a function of temperature.

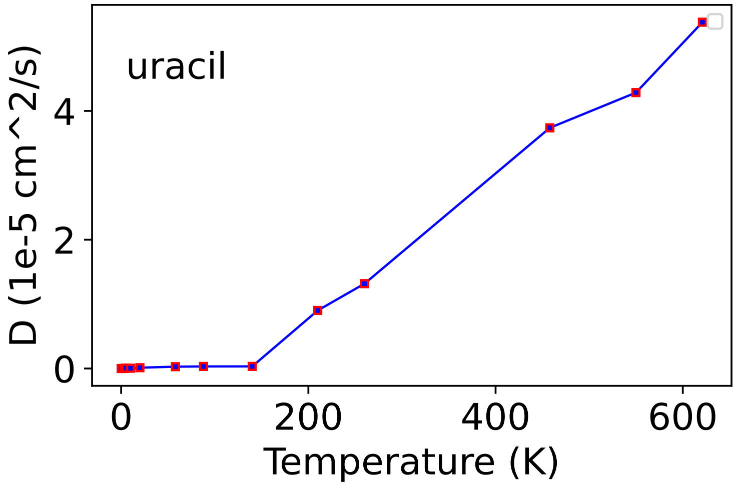

Figure S46: Diffusion as a function of temperature.

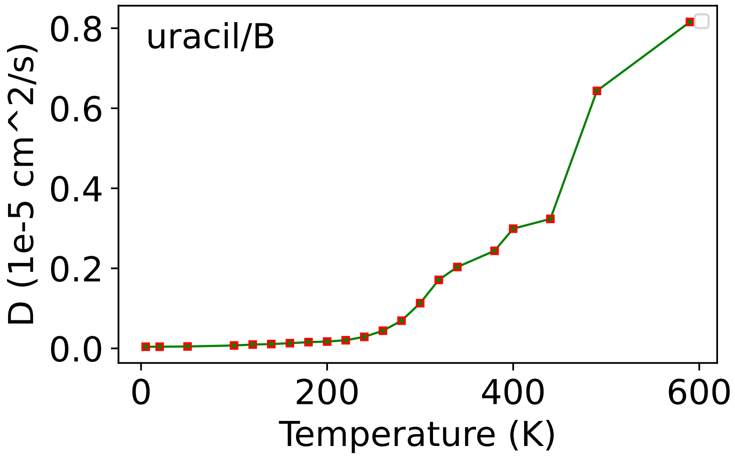

Figure S47: Diffusion as a function of temperature.

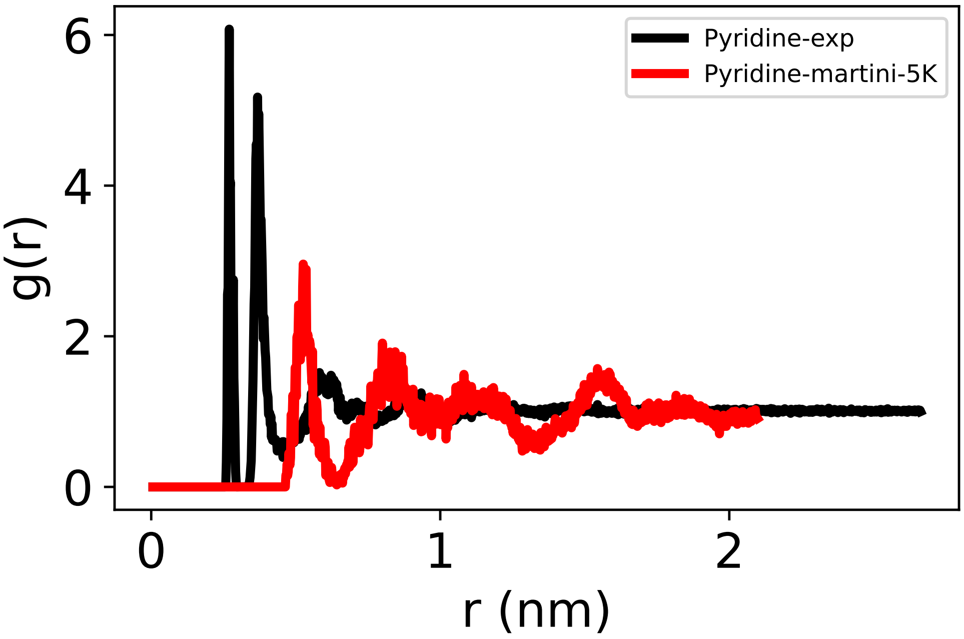

Figure S48: Radial distribution function (RDF) of pyridine at 5 K for both experiment and simulation using the Berendsen barostat.

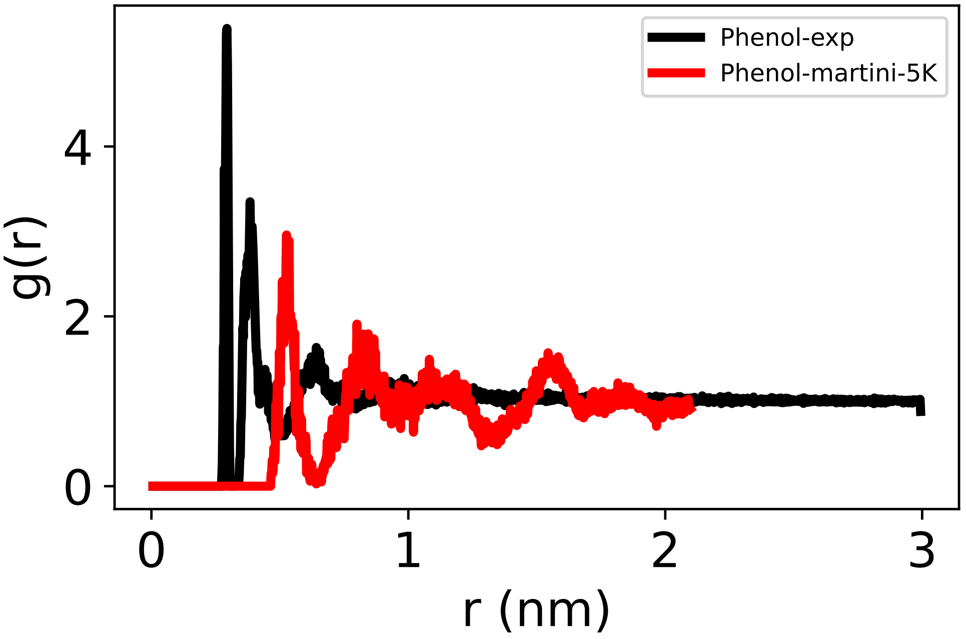

Figure S49: Radial distribution function (RDF) of phenol at 5 K for both experiment and simulation using the Berendsen barostat.

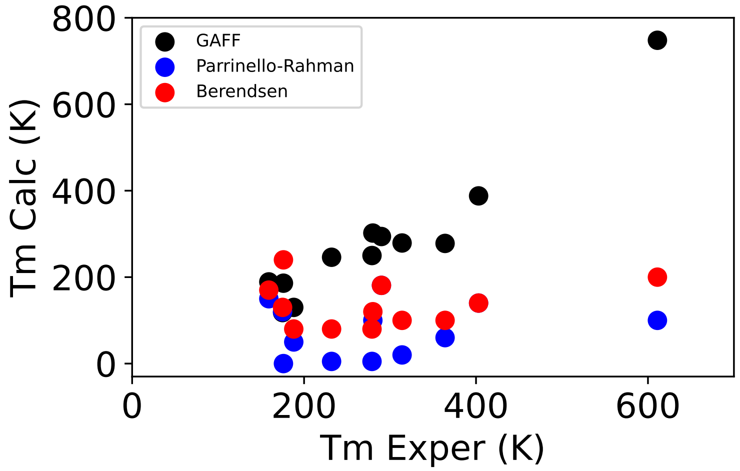

Figure S50: Correlation between experimental melting temperature and simulated melting temperatures for 12 organic compounds (see table 2 in main text).
