# Supplementary material for: Martini on the Rocks: Can a Coarse-Grained Force Field Model Crystals?
Source: J Phys Chem Lett. 2024 Jan 23;15(4):1079–88. doi: 10.1021/acs.jpclett.4c00012 (PMC10839907; doi:10.1021/acs.jpclett.4c00012)
Supplement: Supplementary file 2 — jz4c00012_si_002.pdf [file jz4c00012_si_002.pdf]

jz-2024-00012t.R1

Name: Peer Review Information for "Martini on the Rocks: Can a Coarse-Grained Force Field Model Crystals?"

First Round of Reviewer Comments

Reviewer: 1

Comments to the Author

I think this is an excellent and valuable paper and it should be published with only minor modification pertaining to missing referencing, which I will outline below. The Martini CG forcefield is some sort of a strange and disturbing phenomenon in the field of computational chemistry. It is parameterized in an ad hoc and unphysical way, yet people use it over and over again without questioning it (presumably because it's easy to use). They even claim to simulate a biological "cell" with Martini now. Often authors will just use Martini and make some statement like it "has been extensively validated", which it has not. In that vein, this paper is extremely valuable. I have just one requested revision:

On p. 14 it is stated: "...CG models become less viscous,making interpreting of CG dynamics challenging.28,87" I think ref 23 should be added here.

Reviewer: 2

Comments to the Author

This work presents a comprehensive study of the conformational behavior of aggregating peptides in their crystalline form when simulated under different conditions using the MARTINI 3 force field. The text is well organized and clearly written, presenting a significant volume of simulations. I think it will be of interest to scientists working with CG simulations, which are very popular tools in the field of aggregation processes.

I only have a few suggestions that might be implemented to improve the content.

1- The manuscript is MARTINI-Centric, which might be OK, but might also pass the wrong idea to newcomers that it is the only option. I would suggest including in the introduction some specific mentions to others that have been successfully used to describe aggregation processes (namely,

OPEP, SIRAH, UNRES, etc.). Even the reviews cited are a bit outdated. There are recent reviews addressing pros and cons of CG force fields for proteins.

2- It would be interesting to know if simulations using PR produce actual phase transitions of just distorted cell boxes.

3- The data presented in Table 2 could be better shown by plotting the Tm measured for MARTINI vs Expt and GAFF vs. Expt. Perhaps calculating a correlation could also be informative.

4- Perhaps backmapping the peptide conformations could also be informative and a comparison vs. all-atoms simulations could be done taking advantage of the data already produced by the authors in ref. 45.

Author's Response to Peer Review Comments:

Uppsala, 2024-01-17

Dear Prof. Editor,

thank you for evaluating our manuscript. We were happy to receive constructive referee comments only. Please find our point-by-point responses to those comments below. We hope the manuscript is acceptable in its present form and await your decision.

Sincerely yours,

Najla Hosseini and David van der Spoel, Uppsala
University, Sweden.

EDITORIAL COMMENTS

1. Please include annotated version(s) of your revised publication file(s) with colored textor highlights indicating the revisions that you have made, and upload them as “Supporting Information for Review Only.” Please also upload “clean” copies for publication. (No highlighting, annotations, or colored text permitted.)

ANSWER: Done

2. Author Affiliations: Please place all affiliations on cover page under authors’ list.

ANSWER: Done

3. Author Affiliations: Please include author affiliations in the Supporting-Information

file, matching exactly those of the manuscript file.

ANSWER: Done.

4. Abstract: Shorten the abstract to 150 words or fewer.

ANSWER: Done

5. Headers: Remove the section heading(s) throughout the body of the manuscript (youcan leave Methods, Abstract, and TOC Graphic headings).

ANSWER: Done

6. TOC Graphic: Please resize the TOC graphic per journal guidelines (2 in x 2 in) andmove to the correct position (on the same page as the abstract).

ANSWER: Done

7. References: In both the main file and the supporting information, fix the style of allreferences to use JPCL formatting (check all references carefully).

ANSWER: Reference style was updated.

8. References: Please update the status of 85 and if it is not published then please includea copy of Ref. 85 as Supporting Information for Review.

ANSWER: We removed ref. 85, since the study is still not published.

9. Graphics: One or more of your figures and tables includes a reference citation. Please confirm that this pertains only to data and not the figure itself.

ANSWER: The citations are to scientific papers but the figures are our own.

REVIEWER: 1

Recommendation: This paper is publishable subject to minor revisions noted. Further review is not needed.

Comments: I think this is an excellent and valuable paper and it should be published with only minor modification pertaining to missing referencing, which I will outline below. The Martini CG forcefield is some sort of a strange and disturbing phenomenon in the field of computational chemistry. It is parameterized in an ad hoc and unphysical way, yet people use it over and over again without questioning it (presumably because it's easy to use). They even claim to simulate a biological "cell" with Martini now. Often authors will just use Martini and make some statement like it "has been extensively validated", which it has

not. In that vein, this paper is extremely valuable. I have just one requested revision:

On p. 14 it is stated: ”...CG models become less viscous,making interpreting of CG dynamics challenging.28,87” I think ref 23 should be added here.

ANSWER: Done.

REVIEWER: 2

Recommendation: Reconsider as an article in The Journal of Physical Chemistry A/B/C.

Comments: This work presents a comprehensive study of the conformational behavior of aggregating peptides in their crystalline form when simulated under different conditions using the MARTINI 3 force field. The text is well organized and clearly written, presenting a significant volume of simulations. I think it will be of interest to scientists working with CG simulations, which are very popular tools in the field of aggregation processes. I only have a few suggestions that might be implemented to improve the content.

1- The manuscript is MARTINI-Centric, which might be OK, but might also pass the wrong idea to newcomers that it is the only option. I would suggest including in the introduction some specific mentions to others that have been successfully used to describe aggregation processes (namely, OPEP, SIRAH, UNRES, etc.). Even the reviews cited are a bit outdated. There are recent reviews addressing pros and cons of CG force fields for proteins.

ANSWER: We appreciate your suggestion, and we have added them some references to UNRES simulations as well as a more up-to-date review paper.

2- It would be interesting to know if simulations using PR produce actual phase transitions of just distorted cell boxes.

ANSWER: Thanks for your comment. For all the simulations of organic compounds, the diffusion constant was computed for the last 5 ns of the simulations from the mean square displacement and structures change from the crystal state even at low temperatures using both PR and Berendsen (SI, Fig S24-S47). To better illustrate the transformation of peptides and organic compounds from a crystalline to a liquid state in simulations using Martini3, we presented the radial distribution function (RDF) between backbone beads in the beta sheets. However, the shift in

the calculated RDF and the decrease in RDF height in the simulations, compared to the experimental RDF at both cryo and room temperatures, confirm the melting of the peptide and transformation to a liquid state using both ParrinelloRahman and Berendsen barostats for all three Martini3 variants, see Supporting Information Figs. S48-49 for pyridine and phenol at 5K, S17-22 for NNQQ1 at 100K, and 298 K.

3- The data presented in Table 2 could be better shown by plotting the Tm measured for MARTINI vs Expt and GAFF vs. Expt. Perhaps calculating a correlation could also be informative.

ANSWER: Thanks for your comment. We had already added the correlation plot to the SI, and wrote in the main text that ”There is no or very poor correlation between experimental melting points and those obtained from Martini3 simulations (Fig. S50).”

4- Perhaps backmapping the peptide conformations could also be informative and a comparison vs. all-atoms simulations could be done taking advantage of the data already produced by the authors in ref. 45.

ANSWER: To provide a somewhat quantitative comparison of coarse-grained and allatom simulations we provide a new RDF (Fig. S23), of the atoms that are part of the hydrogen bonds between peptide backbones using the CHARMM36m from our previous AA study. We refer to this figure from the ”Results” section of the paper.
